# Lightweight Ultra‐Strength in AlFeNiTiV Complex Concentrated Alloys via Cu Microalloying‐Driven Lattice Coherency Tuning

**DOI:** 10.1002/advs.202514708

**Published:** 2025-11-08

**Authors:** Hongmei Chen, Weizong Bao, Jie Chen, Tao Hong, Bohua Yu, Xinxin Yang, Ning Ding, Jiayin Chen, Chaoran Wang, Zeyun Cai, Guoqiang Xie

**Affiliations:** ^1^ School of Materials Science and Engineering and Institute of Materials Genome & Big Data Harbin Institute of Technology (Shenzhen) Shenzhen 518055 China; ^2^ Department of Mechanical and Energy Engineering Southern University of Science and Technology Shenzhen 518055 China; ^3^ Research Institute of Physical Sciences in Special Environments Harbin Institute of Technology (Shenzhen) Shenzhen 518055 China; ^4^ State Key Laboratory of Advanced Welding and Joining Harbin Institute of Technology Harbin 150001 China; ^5^ Shenzhen Key Laboratory of New Materials Technology Shenzhen 518055 China

**Keywords:** coherency strengthening, complex concentrated alloys, high specific yield strength, lattice misfit

## Abstract

Next‐generation high‐performance structural materials are required for lightweight design strategies and advanced energy applications. In this work, a custom AlFeNiTiV complex concentrated alloy (CCA) with a combination of ultra‐high compressive strength, over 3.3 GPa, and lightweight, 6.83 g cm^−3^, is developed via Cu microalloying‐driven lattice coherency tuning. The strengthening of the CCA is based on minimal lattice mismatch to achieve maximum coherency strengthening. Maximum precipitation dispersion and alleviating stress concentration at the interface allow the alloy to maintain a macro compressive strain of 11.8%. Meanwhile, it can still maintain excellent yield strength at 600 °C for the low lattice misfit, extremely stable L2_1_ structure, achieving 1311.6 MPa. These findings provide insights into developing lightweight, high‐temperature CCAs through a phase interface modulation strategy.

## Graphical abstract

This study develops a lightweight AlFeNiTiV complex concentrated alloy (density: 6.83 g cm^−^^3^) through Cu microalloying and lattice coherency tuning. The alloy exhibits ultrahigh compressive strength exceeding 3.3 GPa, 11.8% strain, and retains a yield strength of 1311.6 MPa at 600 °C, demonstrating a phase interface strategy for high‐temperature structural materials.

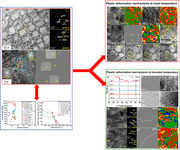

## 1 Introduction

Ordered nanoprecipitates embedded in a disordered matrix are a hallmark of Ni‐,^[^ ^1^ ^]^ Co‐,^[^ ^2^ ^]^ and (FeNi)‐^[^ ^3^ ^]^ based superalloy systems, which have been widely used in a variety of fields such as aerospace,^[^ ^4^ ^]^ marine,^[^ ^5^ ^]^ energy^[^ ^6^ ^]^ and chemical industries^[^ ^7^ ^]^ for their exceptional mechanical strength and high‐temperature creep resistance. The superior properties arose from precipitation hardening, driven by a high density of coherent γ' nanoprecipitations, along with solid‐solution strengthening. However, conventional precipitation strengthening often enhances strength at the expense of ductility.^[^ ^8^, ^9^, ^10^ ^]^ Precipitate size, morphology, and spatial arrangement critically influenced alloy performance.^[^ ^11^ ^]^ Lattice mismatch affected precipitate morphology, growth, and coarsening, ultimately impacting mechanical properties.^[^ ^12^ ^]^ A smaller lattice misfit promoted the formation of fine, high‐density nanoprecipitates, enhancing coherency and strengthening the alloy by impeding dislocation motion.^[^ ^13^ ^]^ In Ni‐based alloys, the present of coherent interfaces effectively obstructs dislocation movement, thereby enhancing strength via co‐lattice interface strengthening.^[^ ^14^ ^]^ Moreover, the uniform dispersion of fine precipitates mitigates localized stress, contributing to improve plasticity.^[^ ^15^, ^16^ ^]^ However, excessive precipitate volume fraction led to coalescence, transitioning from a circular morphology to interconnected structures that degrade performance. Therefore, optimizing the lattice misfit offers a promising strategy to mitigate the strength‐ductility trade‐off, achieving an improved balance between yield strength and ductility.^[^ ^17^ ^]^

The emergence of complex concentrated alloys (CCAs) has significantly expanded the compositional space for alloy design.^[^ ^18^ ^]^ In general, lattice distortion in single‐phase high‐entropy alloys enhances mechanical properties, which can be further improved by the introduction of coherent precipitates. Superalloy‐like microstructures, reinforced by ordered nanoprecipitates, have been identified in certain CCAs, delivering properties comparable to those of commercial Ni‐based superalloys and underscoring their potential as high‐temperature structural materials.^[^ ^19^, ^20^, ^21^ ^]^ Among these, L2_1_‐Ni_2_TiAl‐an ordered variant of the A2 phase, has attracted increasing attention in dual‐phase HEAs due to its excellent specific strength and high‐temperature creep resistance.^[^ ^22^, ^23^ ^]^ Building on the strengthening concepts of ordered nanoprecipitation and eutectic high‐entropy alloys, a series of Al_15_Fe*_x_* Ni_30_Ti_15_V_40−_ *_x_* CCAs was developed in our recent preliminary work.^[^ ^24^ ^]^ The Al_15_Fe_35_Ni_30_Ti_15_V_5_ alloy, featuring a BCC/L2_1_ dual‐phase structure, exhibited both high yield strength and enhanced high‐temperature hardness. However, strengthening via large, incoherent precipitates‐due to their limited slip systems‐often leads to a significant loss in ductility,^[^ ^25^, ^26^ ^]^ with a reported fracture elongation as low as 6.7%, thereby posing challenges for processing and applications. Woo et al.^[^ ^12^ ^]^ demonstrated that the compositional optimization in AlCrFeNiTi CCAs could refine L2_1_‐phase interfaces, as well as precipitate morphology and distribution, thereby markedly improving both mechanical properties and high‐temperature plasticity. These findings suggest that improving lattice coherency through alloy design offers a promising approach to optimizing the mechanical properties of L2_1_‐strengthened CCAs. Nevertheless, precise control over precipitate size and interfacial coherency through compositional tuning remains a great challenge.^[^ ^12^, ^27^, ^28^ ^]^

Cu, as a pivotal alloying element, plays a vital role in microstructural evolution and property modification in CCAs.^[^ ^29^, ^30^ ^]^ In steels, due to its marginal solubility in the α‐Fe matrix, Cu readily participated in the NiAl precipitation process and preferentially occupies Ni sublattice sites.^[^ ^31^ ^]^ The substitution of Ni by Cu effectively reduces the lattice mismatch strain between NiAl precipitates and the α‐Fe (1.24 Å) matrix, influencing precipitation behavior and significantly enhancing material strength. Additionally, Cu addition improves mechanical properties by inducing grain boundary microstructural changes.^[^ ^32^, ^33^ ^]^ Furthermore, as a strong FCC stabilizer, Cu facilitates the formation of FCC phase at high temperatures, thereby enhancing the plasticity of the alloy. Consequently, Cu addition enables the tailoring of interfacial properties and phase constitution, effectively enhancing the performance of both single‐phase and precipitation‐strengthened alloys. To the best of our knowledge, there have been limited studies on lattice mismatch and the underlying strengthening mechanisms in L2_1_‐strengthened CCAs. The advantages of Cu addition on the room‐ and high‐temperature yield strength and deformation behavior of the PBs in dual‐phase CCAs are urged to be evaluated.

Motivated by the aforementioned challenges, a series of (Al_15_Fe_35_Ni_30_Ti_15_V_5_)_100‐x_Cu_x_ CCAs, with Cu doping levels of 0, 0.5, 1.0, and 1.5 at.%, was investigated to alleviate the severe room‐temperature brittleness of the Al_15_Fe_35_Ni_30_Ti_15_V_5_ alloy. Considering the influence of lattice misfit, the physical characteristics of the ordered‐L2_1_ nanoprecipitates‐such as size and number density‐were analyzed, alongside the corresponding strengthening mechanisms and deformation behavior. Though effective coherency strengthening and strain transfer, the (Al_15_Fe_35_Ni_30_Ti_15_V_5_)_99_Cu_1_ alloy, featuring coherent L2_1_ nanoprecipitates, achieved an optimal balance between yield strength and ductility, highlighting its promise for high‐performance structural applications.

## 2 Results

### 2.1 Phase Constitution and Microstructure

**Figure 1:**
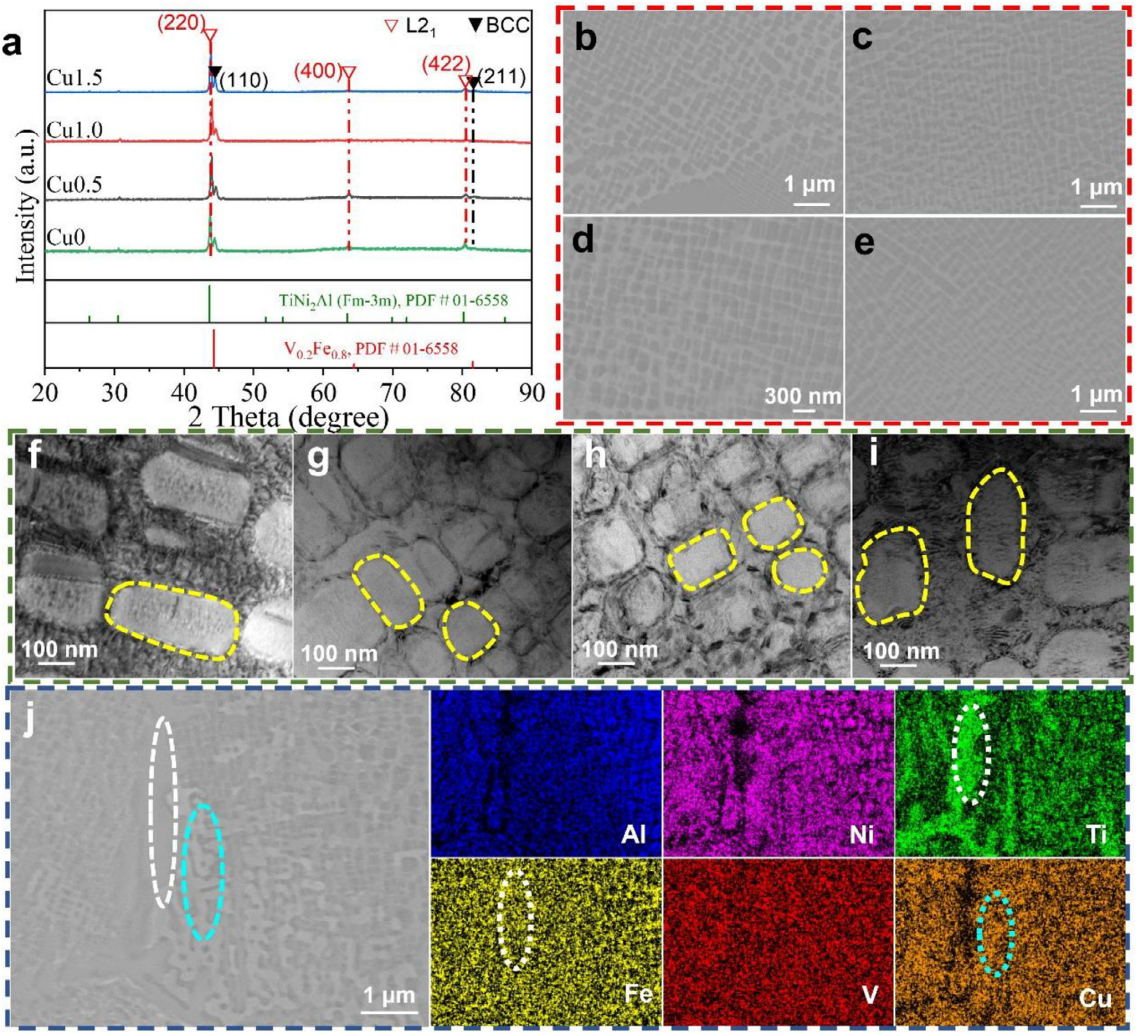
Phase and microstructures of the (Al_15_Fe_35_Ni_30_Ti_15_V_5_)_100‐x_Cu_x_ CCAs: a) XRD patterns of (Al_15_Fe_35_Ni_30_Ti_15_V_5_)_100‐x_Cu_x_ CCAs; BSE‐SEM images of the (Al_15_Fe_35_Ni_30_Ti_15_V_5_)_100‐x_Cu_x_ CCAs: b) Cu0, c) Cu0.5, d) Cu1.0, e) Cu1.5; BFTEM images of the (Al_15_Fe_35_Ni_30_Ti_15_V_5_)_100‐x_Cu_x_ CCAs: f) Cu0, g) Cu0.5, h) Cu1.0, i) Cu1.5; j) EDS mapping of the Cu1.5 alloy.

**Figure** **1**a presents the XRD patterns of the as‐cast (Al_15_Fe_35_Ni_30_Ti_15_V_5_)_100‐x_Cu_x_ alloys with varying Cu contents. All samples exhibit a dual‐phase microstructure comprising BCC and L2_1_ phase. Notably, no additional phases are detected even at the highest Cu content of 1.5 at.%. Satellite peaks corresponding to the ${\left(220\right)}_{L2_{1}}$, ${\left(400\right)}_{L2_{1}}$_,_ and ${\left(422\right)}_{L2_{1}}$ planes of the L2_1_ phase are observed adjacent to (110)_*BCC*_ and (211)_*BCC*_ peaks of the BCC phase, respectively. The XRD patterns obtained from Rietveld refinement (Figure S1, Supporting Information) reveal that shifts in the positions of the ${\left(220\right)}_{L2_{1}}$ and (110)_*BCC*_ peaks, along with alterations in the volume fractions of the two phases. This highlights the significant influence of Cu on the crystal structure of both phases in the alloy system. The lattice mismatch (δ) between two phases is determined by the following equation, which is based on lattice parameters obtained from Rietveld refinement results:^[^ ^12^ ^]^

(1) $$\delta =\frac{2(a_{L2_{1}}-\left(2\times a_{BCC}\right))}{(a_{L2_{1}}+\left(2\times a_{BCC}\right)}$$

where $a_{L2_{1}}$ and *a*_BCC_ represent the lattice parameters of the L2_1_ and BCC phases, respectively. The calculated lattice misfit (δ) decreases from 1.61% (Cu0) to 1.46% (Cu1.0) and then slightly increases to 1.55% (Cu1.5) with increasing Cu content (Table 2).

The mechanical behavior of nanoprecipitate‐strengthened alloys is closely tied to microstructural features such as the precipitate size and morphology. Based on EDS mapping, thermodynamic calculations (Figures S2 and S3, Supporting Information), and our previous work,^[^ ^24^ ^]^ the alloys consist of L2_1_‐rich dendrite cores, while the dendrite periphery exhibits a nanoscale lamellar arrangement of BCC and L2_1_ phases. SEM images (Figure 1b–e) demonstrate the effect of Cu addition on the size and morphology of precipitates at the dendrite periphery. As Cu content increases up to 1.0 at.%, the precipitates‐exhibiting a rounded cuboidal shape‐become finer and more densely distributed, reducing inter‐precipitate spacing. A regular spatial arrangement of precipitates is also observed, attributed to elastic interactions between misfitting particles. These interactions depend on the spacing: repulsive at short range and attractive at longer range, indicating that Cu affects not only the spacing but also the interfacial coherency of precipitates.

To enable quantitative analysis, TEM was employed to characterize the nano‐lamellar BCC/L2_1_ regions. Bright‐field TEM images (BFTEM) and corresponding microstructural features are shown in Figure 1f–i and summarized in **Table** **1**. Moiré fringes are observed at the precipitate‐matrix interface in all alloys, indicating lattice misfit. The average size of L2_1_ nanoprecipitates decreases from 153.6 ± 49.2 nm in Cu0 to 99.0 ± 28.9 nm in Cu1.0, then increases to 124.3 ± 24.2 nm in Cu1.5. Given the geometric asymmetry of the precipitates, their size is reported as equivalent circular diameter. The precipitate morphology evolves from plate‐like shape (Cu0) to rounded cuboidal (Cu1.0), and then reverts to plate‐like (Cu1.5). The geometric asymmetry follows a similar trend, with the aspect ratio approaching 1 in Cu1.0, reflecting morphology changes induced by lattice misfit‐driven growth behavior.

**Table 1:** Microstructure features (average size and width/length of the L2_1_ precipitates of the (Al_15_Fe_35_Ni_30_Ti_15_V_5_)_100‐x_Cu_x_ CCAs.

Alloy	Average size of L2_1_ [nm]	Width/Length
Cu0	153.6 ± 49.2	≈0.394
Cu0.5	131.9 ± 33.0	≈0.834
Cu1.0	99.0 ± 28.9	≈0.879
Cu1.5	124.3 ± 24.2	≈0.639

Cu exhibits higher solubility in the Al‐, Ni‐, and Ti‐enriched L2_1_ phase, causing a leftward shift in its diffraction peaks due to lattice expansion. Concurrently, enhanced Fe partitioning in the BCC phase leads to a similar peak shift (Details regarding Cu partitioning are provided in Note S1, Supporting Information). However, in Cu1.5, due to the limited solubility of Cu in the L2_1_ phase, Cu‐rich precipitates begin to form at grain boundaries^[^ ^34^ ^]^ (Figure 1j). In addition, because of the positive mixing enthalpy of Cu with Fe and Ti, Fe‐ and Ti‐rich precipitates (indicated by white dashed outlines) also appear at grain boundaries. These secondary precipitates contribute to rightward shifts in the diffraction peaks of both phases, suggesting local compositional variations and potential phase evolution.

**Figure 2:**
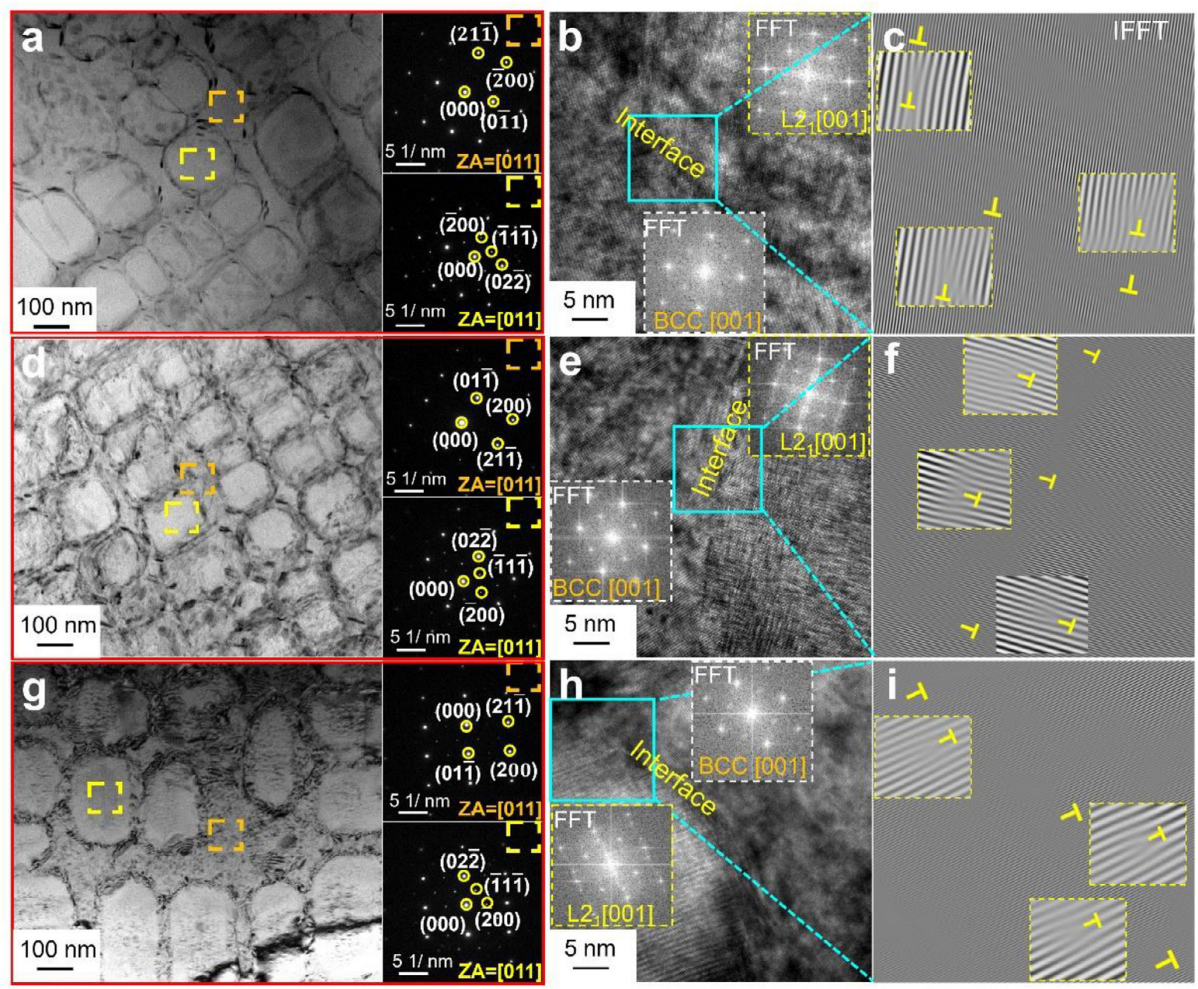
BFTEM images and the corresponding SAEDs of the (Al_15_Fe_35_Ni_30_Ti_15_V_5_)_100‐x_Cu_x_ CCAs: a) Cu0.5, d) Cu1.0, g) Cu1.5, HRTEM images of the alloy, showing the dual‐phase interface of L2_1_/BCC; The upper and lower insets show the FFT of the L2_1_ and BCC phases, respectively: b) Cu0.5,e) Cu1.0, h) Cu1.5; the corresponding inverse FFT from the magnified‐HRTEM images: c) Cu0.5, f) Cu1.0, i) Cu1.5.

To obtain detailed structural information on the phases of the CCAs, TEM analysis was performed, as shown in **Figure** **2**. Selected‐area diffraction (SAED) patterns (insets of Figure 2a,d,g) confirm that the matrix is BCC phase, while the nanoprecipitates exhibit an ordered L2_1_ structure, as evidenced by the characteristic superlattice reflections of ${\left(022\right)}_{L2_{1}}$ and ${\left(111\right)}_{L2_{1}}$. Furthermore, SAED patterns of the BCC phase consistently exhibit superlattice reflections, indicating the presence of a more finely ordered precipitate phase within the BCC matrix. High‐resolution transmission electron microscope (HRTEM) images of the L2_1_/BCC phase interfaces are presented in Figure 2b,e,h, along with the corresponding fast Fourier transform (FFT) for each phase. A high density of edge dislocations is observed at the phase boundaries, as highlighted in the inverse FFT (IFFT) magnified images in Figure 2c,f,i. These dislocations suggest substantial strain accumulation and interfacial mismatch between the L2_1_ and BCC phases.

The lattice misfit between the precipitate and matrix is also calculated from the spacing of the misfit dislocations observed in the HRTEM images. As can be seen from the XRD results, the lattice parameter of the BCC matrix is smaller than that of L2_1_ precipitate (see **Table** **2**). This leads to the formation of an extra‐half plane of edge dislocations of the interface. The lattice misfit calculated from the HRTEM images is inversely related to the spacing of the dislocations as follows:^[^ ^12^ ^]^

(2) $$\delta =\frac{\left|b\right|}{D}$$

where, b = ((d_hkl_, L2_1_/2) + d_hkl_, BCC)/2 is the edge component of the Burgers vector, and D is the misfit dislocation spacing. To determine the misfit dislocation spacings in the present alloys, we selected average‐sized precipitates and acquired the HRTEM images along the interface of the precipitates ((Figure 2c,f,i) (The HRTEM images of Cu0 were provided in Figure S4, Supporting Information). The resultant δ_HR_ with the change in the Cu is presented in Table 2. The lattice misfit calculated from the dislocation spacing of the precipitates is comparable to the lattice misfit determined from the XRD results. Therefore, the estimated lattice misfit from the XRD results is acceptable despite the smaller size of the specimens.

**Table 2:** Lattice constant and lattice misfit between the L2_1_ and BCC phases of the (Al_15_Fe_35_Ni_30_Ti_15_V_5_)_100‐x_Cu_x_ CCAs determined by HRTEM.

Alloys	XRD (110)_*BCC*_ & ${\left(220\right)}_{L2_{1}}$ diffraction				HRSTEM dislocation spacing	δ_HR_[%]
	*a_BCC_* [Å]	$a_{L2_{1}}\left[Å\right]$	δ_XRD_[%]			
Cu0	2.884	5.862	1.61	//<110>	12.15 ± 1.24	1.69
Cu0.5	2.891	5.869	1.51	//<110>	13.61 ± 3.04	1.51
Cu1.0	2.894	5.872	1.46	//<110>	14.95 ± 2.32	1.38
Cu1.5	2.886	5.862	1.55	//<110>	13.46 ± 3.42	1.53

### 2.2 Mechanical Properties

**Figure 3:**
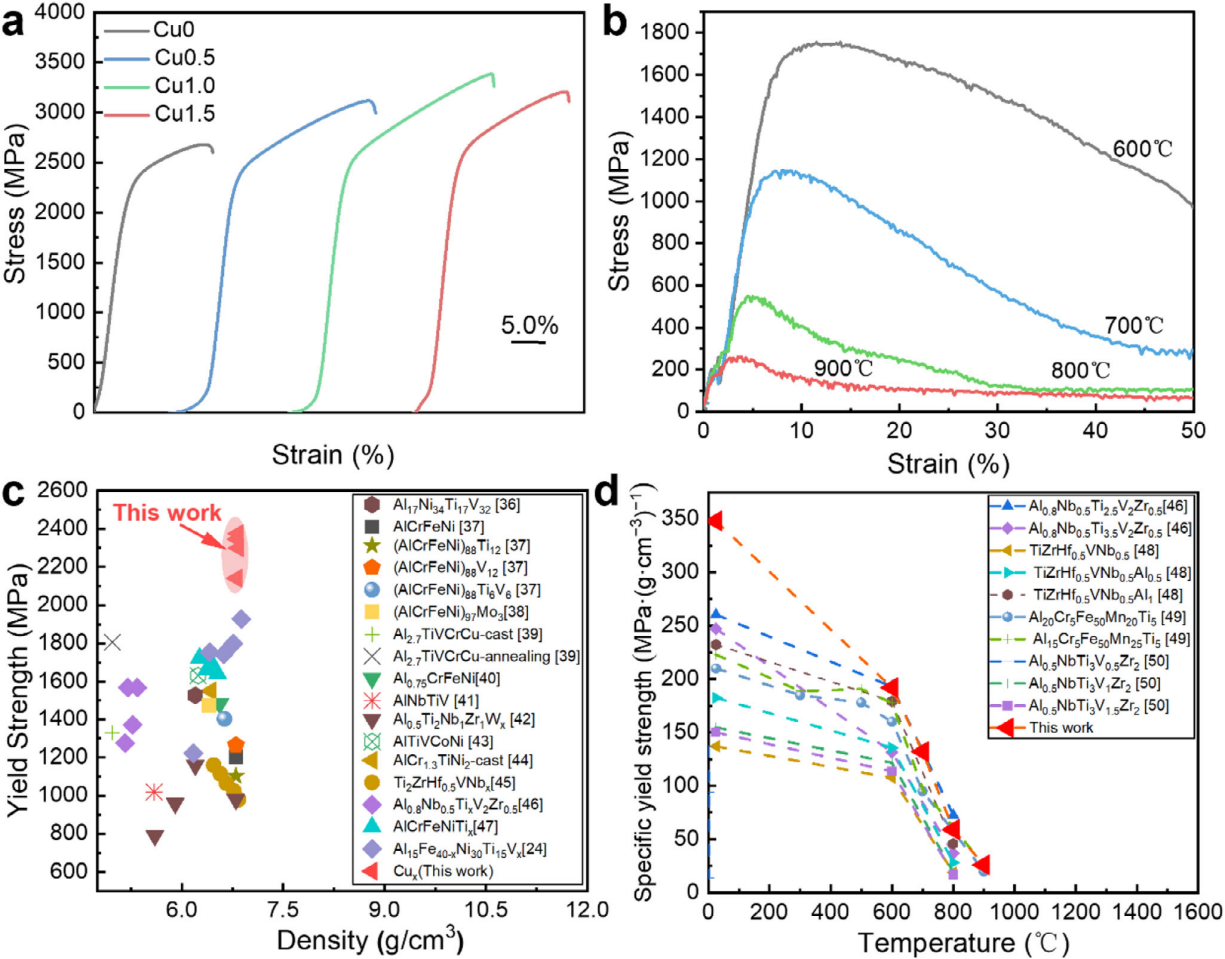
Mechanical properties of the (Al_15_Fe_35_Ni_30_Ti_15_V_5_)_100‐x_Cu_x_ CCAs: a) compression stress–strain curves of (Al_15_Fe_35_Ni_30_Ti_15_V_5_)_100‐x_Cu_x_ CCAs; b) compressive engineering stress–strain curves at various temperatures of the Cu1.0 CCAs; c) yield strength and density of the (Al_15_Fe_35_Ni_30_Ti_15_V_5_)_100‐x_Cu_x_ CCAs compared with other representative alloys;^[^ ^24^, ^36^, ^37^, ^38^, ^39^, ^40^, ^41^, ^42^, ^43^, ^44^, ^45^, ^46^, ^47^ ^]^ d)SYS as a function of testing temperature of this alloy in comparison with other representative alloys.^[^ ^46^, ^48^, ^49^, ^50^ ^]^

We first report the mechanical properties at room temperature. Compared with the base alloy Al_15_Fe_35_Ni_30_Ti_15_V_5,_ which exhibits a yield strength (𝜎_y_) of ≈2140.9 ± 50.1 MPa, an ultimate compressive strength (𝜎_UCS_) of 2699.7 ± 28.0 MPa, and a fracture strain (ɛ_f_) of 6.7 ± 0.4%, the Cu1.0 alloy shows significantly enhanced performance. Specifically, 𝜎_y_ increases by 75%, and the ductility improves to 11.8 ± 0.2%, as shown in **Figure** **3**a and summarized in Table S2 (Supporting Information). Notably, the Cu1.0 alloy exhibits pronounced strain hardening, resulting in an exceptional compressive strength of 3371.6 ± 80.4 MPa. The solid solution strengthening effect arising from the incorporation of Cu into BCC and L2_1_ phases contributes to the increased yield strength. Furthermore, the reduction of the lattice misfit enhances coherency strengthening.^[^ ^12^ ^]^ In addition, the increased density of L2_1_ precipitates helps to alleviate stress localization during loading, thereby improving plasticity.^[^ ^35^ ^]^ However, further Cu addition leads to the formation of a brittle Laves phase and increased lattice misfit between the two primary phases, which in turn reduces both the yield strength and ductility of the Cu1.5 alloy. To evaluate the high‐temperature mechanical behavior, compression tests were conducted at 600, 700, 800, and 900 °C for the Cu1.0 alloy (Figure 3b; Table S3, Supporting Information). Remarkably, the alloy retains a high yield strength of 1311.6 ± 15.2 MPa at 600 °C and exhibits excellent plasticity at elevated temperatures. The combination of 𝜎_y_, 𝜎_UCS_, low density and high specific yield strength (SYS) at elevated temperature clearly distinguishes the Cu1.0 alloy from previously reported alloy systems. This is further illustrated by the comparative data on compressive properties in Figures 3c,d (details of the literature sample are provided in Table S4, Supporting Information), which highlight the superior mechanical performance of the Cu1.0 alloy relative to previous developed eutectic HEAs (EHEAs), lightweight HEAs (LWHEAs).

## 3 Discussion

### 3.1 Strengthening Mechanism at Room Temperature

The yield strength of metallic materials is typically considered as the sum of several independent strengthening mechanisms: lattice friction strength (σ_0_), solid solution hardening (Δσ_*ss*_), grain boundary strengthening (Δσ_*GB*_), precipitation strengthening (Δσ_*ppt*_) and dislocation strengthening (Δσ_*dis*_), which could be expressed as:

(3) $$\sigma_{y,RT}=\sigma_{0}+\Delta \sigma_{ss}+\Delta \sigma_{GB}+\Delta \sigma_{ppt}+\Delta \sigma_{dis}$$

where σ_0_ is estimated to be 206 MPa for pure iron.^[^ ^12^ ^]^ Solid solution strengthening, Δσ_*ss*_, which depends on the concentration of solute elements in the matrix, contributes ≈187 MPa (details calculations are provided in Note S2, Supporting Information). Grain boundary strengthening, Δσ_*GB*_, derived using the Hall–Petch relationship, contributes ≈51 MPa (see Note S3, Supporting Information). Precipitation strengthening, a key mechanism for enhancing σ_*y*_ in both conventional alloys and CCAs,^[^ ^51^ ^]^ is governed by the precipitate type, size, and distribution.^[^ ^52^ ^]^ In the present study, two distinct interaction mechanisms exist between the nanoprecipitates and dislocations. The nanoprecipitates (≈3 nm) in the BCC matrix hinder dislocation motion via the dislocation shearing mechanism, whereas the coherent L2_1_ nanoprecipitates (≈99 nm) located in the inter‐dendritic regions impede dislocation motion via the Orowan mechanism. Moreover, coherency strengthening Δσ_*coh*_, the dominant mechanism at room temperature, is inversely proportional to the precipitate size (see Note S4, Supporting Information). Therefore, the Cu1.0 alloy, which possesses the smallest precipitate size and lowest lattice mismatch, achieves the most effective coherent strengthening. The precipitation strengthening contribution in the (Al_15_Fe_35_Ni_30_Ti_15_V_5_)_99_Cu_1_ CCAs can be calculated as: Δσ_*ppt*_ = σ_*y*,*RT*_‐(σ_0_+ Δσ_*ss*_+Δσ_*GB*_) = 1931 MPa. This result confirms that precipitation strengthening is the primary mechanism contributing to the yield strength in this alloy system. Furthermore, the superior room‐temperature yield performance of the Cu1.0 alloy can be attributed to improved lattice coherency and the refinement of nanoprecipitates induced by Cu addition.

### 3.2 Plastic Deformation Mechanisms at Room Temperature

**Figure 4:**
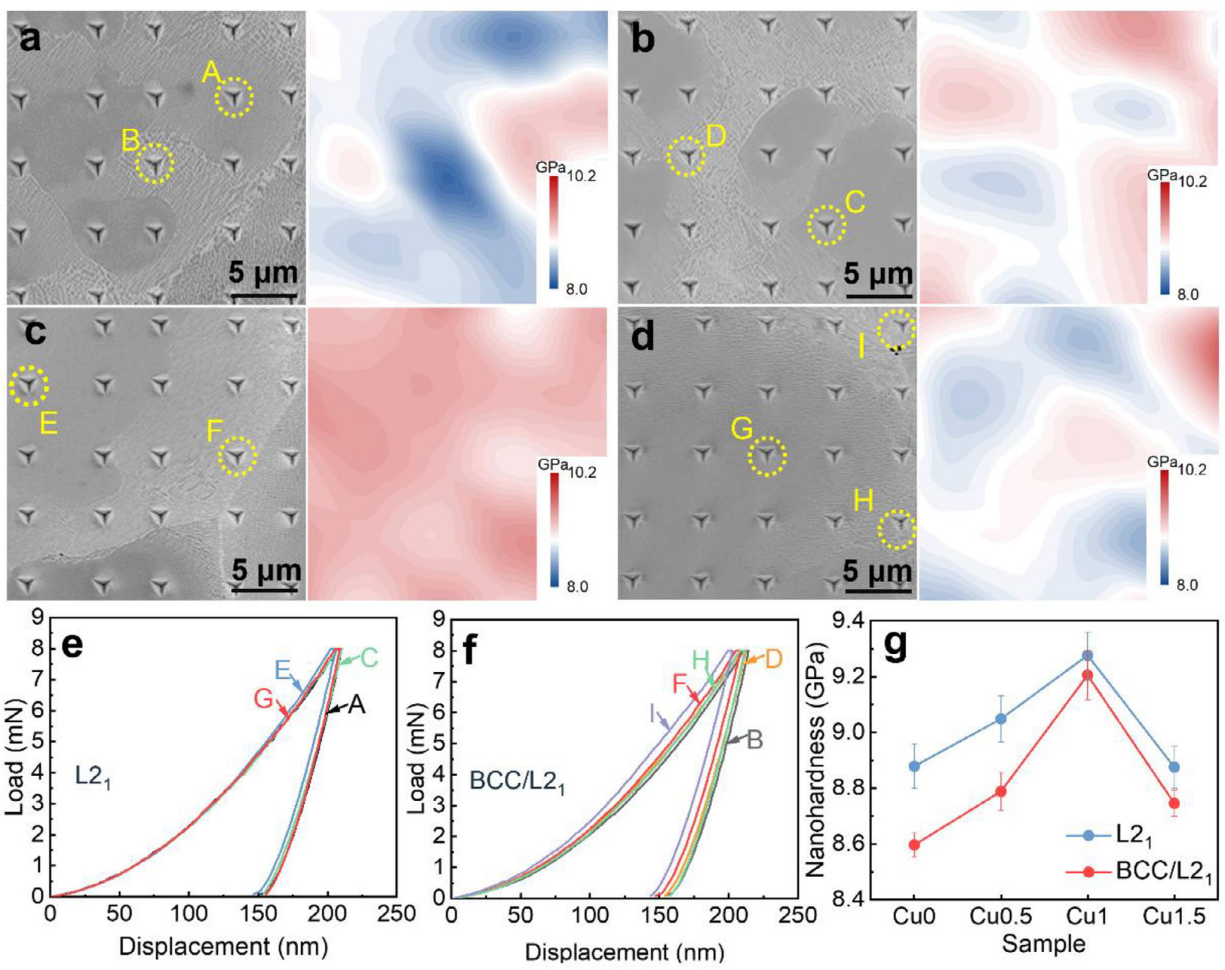
Nanoindentation topographic and the corresponding hardness map of the (Al_15_Fe_35_Ni_30_Ti_15_V_5_)_100‐x_Cu_x_ CCAs: a) Cu0, b) Cu0.5, c) Cu1.0, d) Cu1.5; e,f) typical load‐displacement curves of the L2_1_ and BCC/L2_1_ phases; g) average nano‐hardness for the L2_1_ and BCC/L2_1_ phases.

A nanoindentation test was conducted to continuously record the load‐displacement behavior at the nanometer scale, enabling accurate assessment of the mechanical properties of distinct microstructural regions. To investigate the influence of Cu addition on the mechanical behavior of various phase regions, quasi‐static nanoindentation tests were performed on the L2_1_ phase and BCC/L2_1_ lamellar regions. A total of 25 indentation points were tested in each region, with a spacing of 5 µm between adjacent points. **Figure** **4**a–d present nanoindentation topographic images and the corresponding hardness maps. The hardness distribution strongly correlates with the phase distribution: the bright regions (L2_1_ phase) exhibit significantly higher hardness than the gray BCC/L2_1_ lamellar regions. This clearly highlights mechanical incompatibility between the hard L2_1_ phase and the softer BCC/L2_1_ regions. This heterogeneous mechanical response is further supported by the distinct load‐displacement curves obtained from the two regions, as shown in Figure 4e,f. Among all compositions, the Cu1.0 alloy exhibits the highest nanoindentation hardness (Figure 4g). The variation in hardness within the L2_1_ regions is primarily attributed to the solid solution strengthening induced by Cu incorporation. In contrast, the enhanced hardness in the BCC/L2_1_ regions is attributed to improved co‐lattice strengthening, resulting from the reduced lattice mismatch between the two phases.

**Figure 5:**
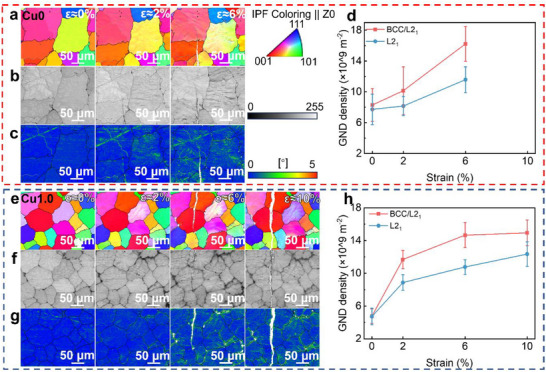
Microstructure evolution of the Cu0/Cu1.0 alloy at different strain amplitudes: a,e) Electron backscatter diffraction (EBSD) inverse‐pole figure (IPF) maps for Cu0 and Cu1.0 alloy sat the strain amplitudes of 0%, 2%, 6%, and 10%, respectively; b,f) the corresponding BC maps; c,g) the corresponding kernel average misorientation (KAM) distribution maps; d,h) variations of average geometrically necessary dislocations (GND) densities in the BCC/L2_1_ and L2_1_ phase regions differentiated on the basis of BC maps, respectively. The error bars represent the corresponding standard deviation, which are obtained from 3 independent EBSD mappings on the regions with identical local strain levels. The inset shows the variation in the average misorientation of the alloy with increasing plastic strain.

The surface morphologies of Cu0 and Cu1.0 alloys at various deformation stages reveal that most grains undergo inhomogeneous plastic deformation, primarily governed by dislocation slip (Figure S6, Supporting Information). To further elucidate the deformation behavior, microstructural evolution at different strain levels was analyzed using EBSD, as shown in **Figure** **5**a,e. The equiaxed grain structure remains largely intact throughout deformation, reflecting high structural stability in both Cu0 and Cu1.0 alloys. Prior to loading (ε≈0%), the uniform color within each grain suggests consistent crystallographic orientation. At ε≈2%, subtle color changes indicate the onset of grain rotation and plastic deformation. With increasing strain, grain rotation intensifies. At 6% strain for Cu0 and 10% strain in Cu1.0, significant intragranular color variations appear, reflecting local lattice rotation in multiple directions and indicating non‐uniform plastic deformation.

Due to the similar lattice structures of the BCC and L2_1_ phases, distinguishing them via EBSD is challenging. However, EBSD‐based approaches leverage differences in lattice defect densities‐reflected in the image quality (IQ) and band contrast (BC) ‐ to differentiate similar phases.^[^ ^53^ ^]^ BC maps provide morphological contrast information that enables phase distinction based on defect density. Accordingly, L2_1_ and BCC/L2_1_ regions in this study were identified using BC maps. Plastic strain distribution was further analyzed using kernel average misorientation (KAM) maps (Figure 5c,g) derived from EBSD data. KAM values correlate with the density of geometrically necessary dislocations (GNDs), which serve as indicators of localized plastic strain.^[^ ^54^ ^]^ Higher KAM values correspond to higher GND densities and higher local plastic deformation. To quantify plastic strain accommodation in each phase, the average GND density was calculated at different strain levels for both Cu0 (Figure 5d)and Cu0 alloy (Figure 5h). At low strain, the BCC/L2_1_ phase region in the Cu1.0 alloy exhibits relatively higher KAM values than the L2_1_ phase (Figure 5h). From 0% to 6% strain, GND density in the BCC/L2_1_ phase region increases rapidly from 4.76 × 10^9^ to 14.66 × 10^9^ m^−2^, whereas in the bulk L2_1_ phase region, it increases from 4.71 × 10^9^ to 10.76 × 10^9^ m^−2^. With further deformation, GND density in the bulk L2_1_ phase region surpasses that in the BCC/L2_1_ phase region, eventually resulting in a comparable value. This evolution enables more uniform plastic deformation at 10% strain in the Cu1.0 alloy (Figure 5h). In contrast, the Cu0 alloy exhibits a pronounced mismatch in GND densities between the bulk L2_1_ phase and BCC/L2_1_ phase regions, suggesting poorly coordinated plastic deformation. The increased misorientation between BCC/L2_1_ and L2_1_ phases with increasing strain in the Cu1.0 alloy also indicates strain delocalization during compression deformation.

**Figure 6:**
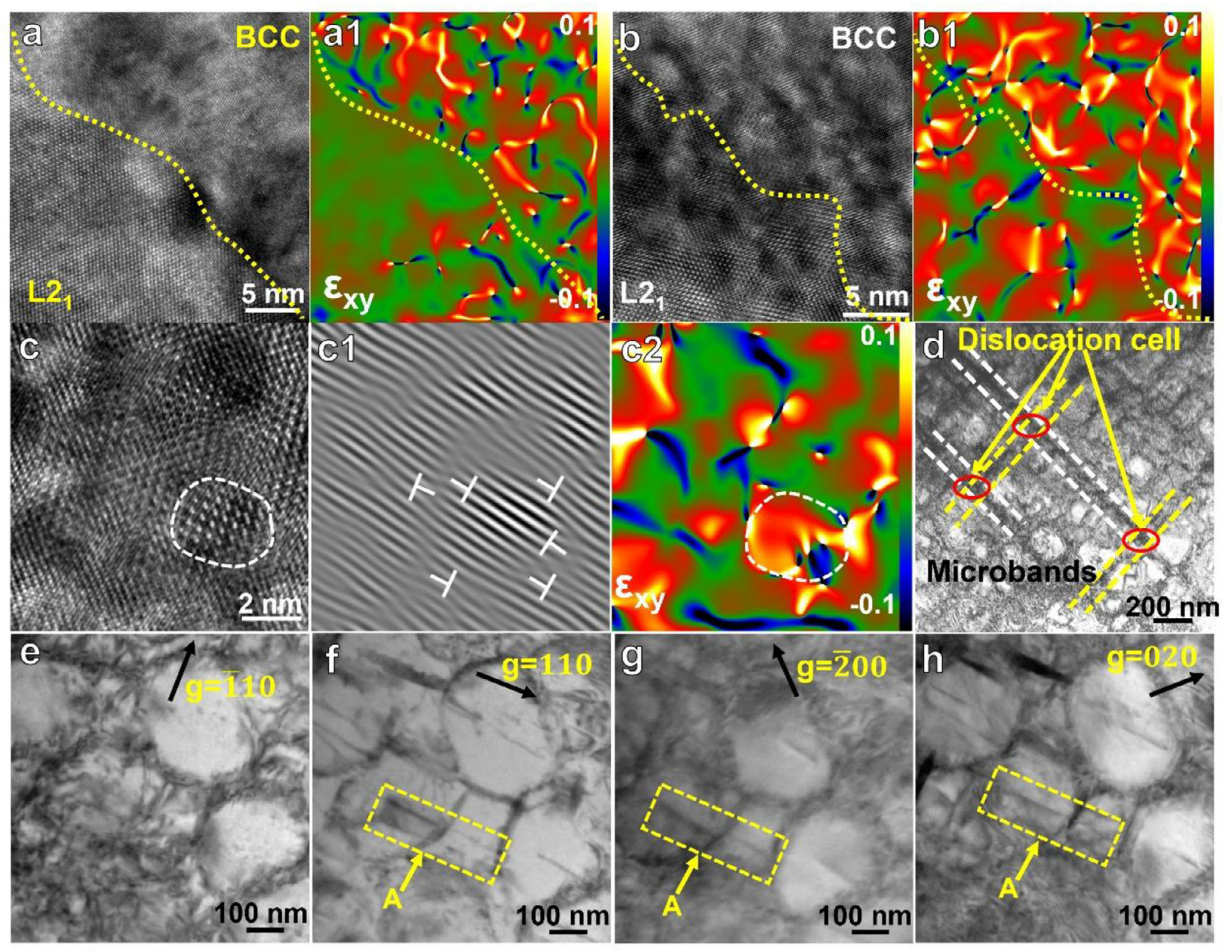
Activation of multiple deformation mechanisms in the compressed‐deformed. Cu1.0 alloy: a,a1,b,b1) HRTEM image and the corresponding GPA strain map of the alloy before and after deformation, showing the dual‐phase interface of L2_1_/BCC; c–c2) HRTEM image, IFFT micrograph and GPA for the BCC phase after deformation; d) the BFTEM image of the deformation dislocation substructure; e–h) a series of BF images detailing dislocation “A” interaction between the L2_1_ and BCC phases from the dislocation.

To further investigate the underlying deformation mechanisms, transmission electron microscope (TEM) analysis was conducted on the Cu1.0 alloys at maximum strain. High‐resolution TEM (HRTEM) images of the dual‐phase interface before and after 10% compression, along with corresponding lattice strain maps obtained from Geometrical phase analysis (GPA), are shown in **Figure** **6**a,a1,b,b1. Prior to deformation, the GPA maps reveal non‐uniform strain distributions within the lattice, attributed to the high density of L2_1_ nanoprecipitates embedded in the BCC matrix. After deformation, significant strain accumulation is observed within the L2_1_ nanoprecipitates in the BCC matrix, indicating that the ≈3 nm L2_1_ precipitates effectively impede dislocation motion through the dislocation shearing mechanism. Fast Fourier transform (FFT) patterns and GPA analyses were performed on both the BCC matrix phase and the L2_1_ nanoprecipitate region (Figure 6c–c2). Due to the difference in lattice constants between the two phases, pronounced atomic‐level strain fluctuations are present near the precipitate‐matrix interface. The severely distorted region (outlined by a dashed line) corresponds to the interface between the BCC matrix and the L2_1_ nanoprecipitates. In this deformation stage, multiple microbands form in various directions within the alloy, as shown in Figure 6d. Dislocations within these microbands interact and entangle, forming dense dislocation networks that impede dislocation mobility and contribute to strain hardening.

For detailed dislocation analysis, regions with low dislocation density were selected. In particular, dislocation “A,” marked by the yellow box, was imaged under various diffraction conditions, as shown in Figure 6e–h. The dislocation is visible under g = [111], [$\bar{2}$00], and [020] conditions, while it is rendered invisible under g = [$\bar{1}$10], from which the Burgers vector is identified as b = 1/3a<111>. Additionally, a clear one‐to‐one correspondence between dislocations on either side of the interface is observed, confirming the occurrence of slip transmission across the BCC/L2_1_ boundary.

**Figure 7:**
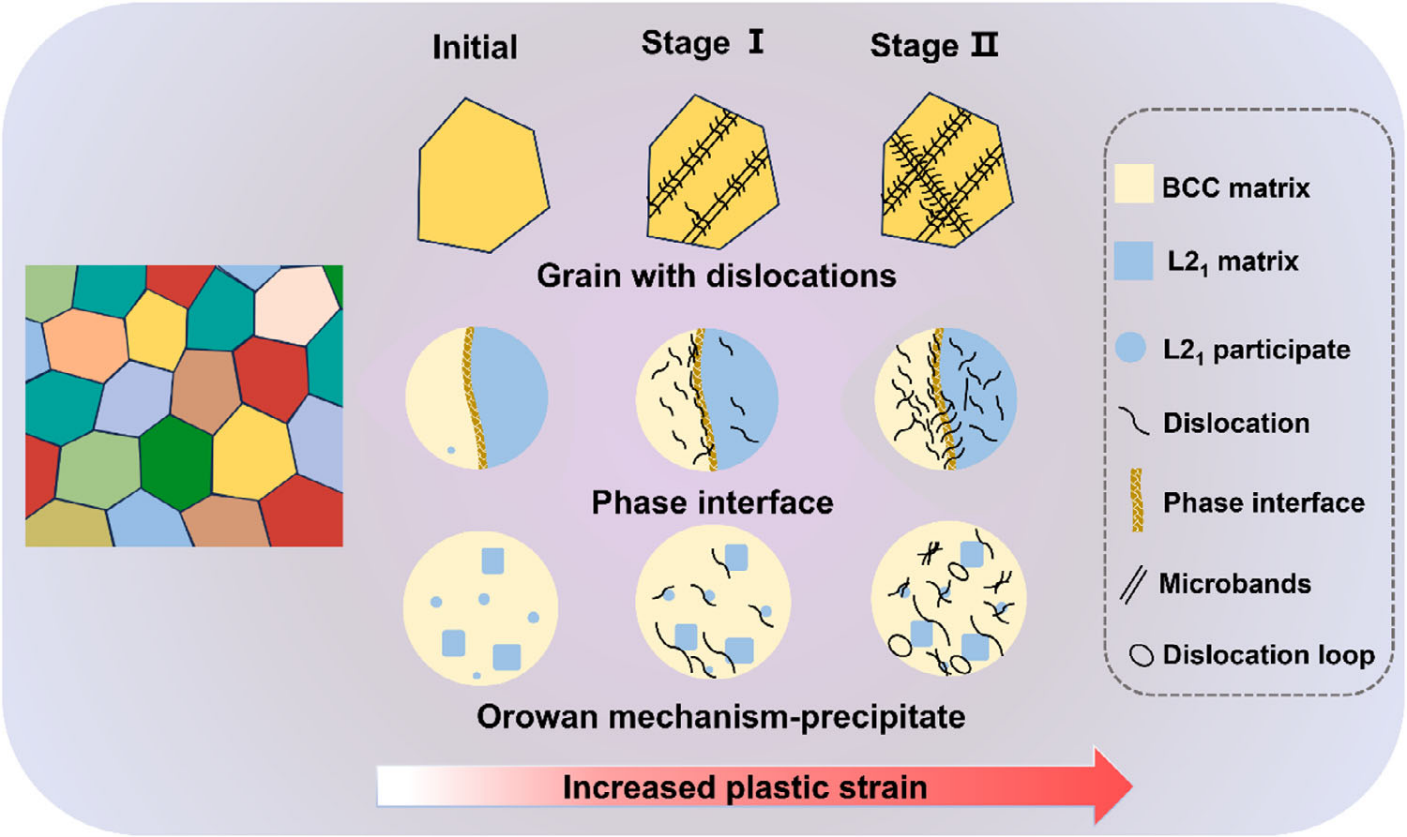
Schematic of the initial microstructures and deformation mechanism of Cu1.0 alloy during the compression deformation process.

A schematic summary of the microstructural evolution in the Cu1.0 alloy is presented in **Figure** **7**. Plastic deformation is primarily accommodated by the formation of narrow, sparse slip microbands, which progressively increase in number with accumulating strain. Dislocations are preferentially nucleated in the softer BCC phase and then transmitted into the harder L2_1_ phase across the coherent interface. This slip transfer mechanism effectively alleviates interfacial stress concentrations and facilitates coordinated deformation between the two phases. As plastic strain increases, the dislocation density rises in both phases, leading to the development of dislocation‐rich microbands near the interface, contributing to strain‐hardening. Moreover, the interaction between dislocations and nanoscale L2_1_ precipitates in the BCC region promotes the formation of dislocation tangles, further enhancing the strain‐hardening response of the alloy.

### 3.3 Plastic Deformation Mechanisms at Elevated Temperature

Experimental results demonstrate that the outstanding mechanical properties of the present Cu1.0 alloy, particularly at high temperatures, originate from its unique strengthening mechanisms.

**Figure 8:**
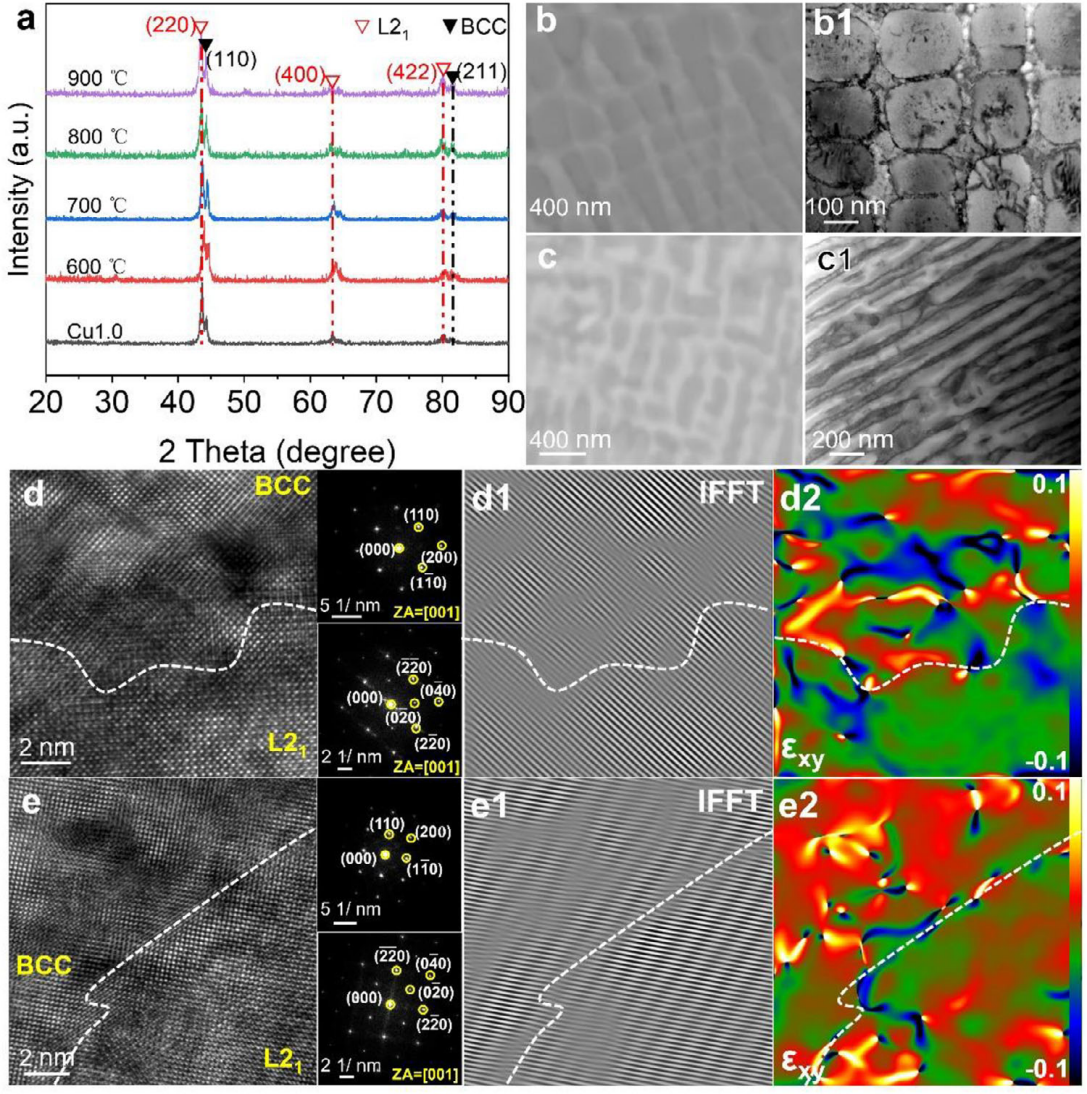
Phase and microstructures of the Cu1.0 alloy deformed at elevated temperatures: a) XRD patterns; BSE‐SEM and BFTEM images the Cu1.0 alloy deformed at different temperatures: b,b1) 600 °C, c,c1) 900 °C; HRTEM images, IFFT micrograph and GPA for the interfacial regions after deformation; The upper and lower insets show the SAEDs of the BCC and L2_1_ phases, respectively: d–d2) 600 °C, e–e2) 900 °C.

The thermal stability of phases and microstructure is critical for the mechanical performance of materials at elevated temperatures and serves as a key indicator of the long‐term serviceability of high‐temperature structural materials. XRD patterns of the Cu1.0 alloy compressed to peak strain at different temperatures (**Figure** **8**a) reveal an identical phase composition to that at room temperature, indicating the absence of phase transitions during high‐temperature compression. SEM and BFTEM images (Figure 8b,b1) show that the L2_1_ nanoprecipitates maintain their original morphology after deformation at 600 °C, despite experiencing slight coarsening (189.2 ± 20.5 nm). Furthermore, EBSD analysis confirms no evidence of recrystallization at this temperature (Figure S7, Supporting Information). These findings collectively affirm the exceptional microstructural stability of the Cu1.0 alloy during compression at 600 °C. In contrast, the microstructure after compression at 900 °C (Figure 8c,c1) reveals significant coarsening of the L2_1_ nanoprecipitates, which adopt an elongated, strip‐like morphology under compressive stress. BFTEM observations indicate that dislocations accumulate predominantly at the L2_1_/BCC phase interfaces and within the BCC matrix, suggesting the occurrence of nonuniform deformation.

The lattice misfit increases with increasing temperature due to the higher thermal expansion coefficient of L2_1_ precipitate than that of the BCC matrix. An excessively large misfit can trigger abrupt changes in interfacial properties, thereby degrading the high‐temperature performance of the alloy. HRTEM characterization of the L2_1_/BCC interfaces after compression at 600 °C and 900 °C was conducted, complemented by IFFT and GPA (Figure 8d–d2,e–e2). The accumulation of dislocations at these interfaces and within the BCC phase corresponds to regions of high localized strain. The lattice parameters of the two phases were obtained from HRTEM (Table S9, Supporting Information), and the corresponding lattice misfits at 600 and 900 °C were calculated to be 1.77% and 2.57%, respectively, using Equation (1). The retained lattice coherency at the L2_1_/BCC interface at 600 °C is a key contributor to the alloy's superior performance, providing significant coherency strengthening and positioning the Cu1.0 alloy as an ideal candidate for intermediate‐temperature service.

## 4 Conclusion

In summary, Cu microalloying achieves a synergistic enhancement in both strength and plasticity of Al_15_Fe_35_Ni_30_Ti_15_V_5_ CCA, providing new insights into phase‐boundary strengthening mechanisms and deformation behavior. The key conclusions are as follows:

- Cu addition, owing to the differing solid solubility in the L2_1_‐Ni_2_AlTi phase and (Fe,V)‐rich BCC phase, initially enhances and then reduces the interfacial coherency of the L2_1_ nanoprecipitates. The Cu1.0 alloy exhibits the finest L2_1_ nanoprecipitates size and the lowest lattice mismatch with the matrix.

ii) Cu microalloying effectively balances the strength‐ductility trade‐off in the AlFeNiTiVCu alloy system. The Cu1.0 alloy achieves a high yield strength of 2375.2 MPa and a plastic strain of 11.8%. Its specific yield strength is as high as 347.8 MPa (g cm^−3^)^−1^, demonstrating its superior mechanical performance. The enhanced strength stems from precipitation strengthening, while improved lattice compatibility promotes synergistic coherency strengthening and Orowan dislocation looping. High‐density L2_1_ nanoprecipitates alleviate stress concentration, enhancing ductility.
iii) The Cu1.0 alloy exhibits exceptional high‐temperature mechanical performance, maintaining a specific yield strength of 192 MPa (g cm^−3^)^−1^ at 600 °C. This is attributed to the retained lattice coherency between the L2_1_ precipitates and the matrix at 600 °C, indicating excellent high‐temperature stability.

## 5 Experimental Section

Experimental Section can be found in the Supplementary Materials (see Note S5, Supporting Information).

## Conflict of Interest

The authors declare no conflict of interest.

## Supporting information



Supporting Information

## Data Availability

Research data are not shared.
