# Supplementary material for: Lightweight Ultra‐Strength in AlFeNiTiV Complex Concentrated Alloys via Cu Microalloying‐Driven Lattice Coherency Tuning
Source: Adv Sci (Weinh). 2025 Nov 8;13(6):e14708. doi: 10.1002/advs.202514708 (PMC12866750; doi:10.1002/advs.202514708)
Supplement: Supplementary file 1 — Supporting Information [file ADVS-13-e14708-s001.docx]

Supporting Information

**Lightweight Ultra-strength in AlFeNiTiV Complex Concentrated Alloys via Cu Microalloying-driven Lattice Coherency Tuning**

Hongmei Chen^a^, Weizong Bao^b, *^, Jie Chen^a^, Tao Hong^a^, Bohua Yu^a^, Xinxin Yang^a^, Ning Ding^a^, Jiayin Chen^a^, Chaoran Wang^a^, Zeyun Cai^c, *^, Guoqiang Xie^a, d, e, *^

*a School of Materials Science and Engineering, and Institute of Materials Genome & Big Data, Harbin Institute of Technology (Shenzhen), Shenzhen, 518055, China*

*b Department of Mechanical and Energy Engineering, Southern University of Science and Technology, Shenzhen, 518055, China*

*c* *Research Institute of Physical Sciences in Special Environments, Harbin Institute of Technology (Shenzhen), Shenzhen, 518055, China*

*d State Key Laboratory of Advanced Welding and Joining, Harbin Institute of Technology, Harbin, 150001, China*

*e Shenzhen Key Laboratory of New Materials Technology, Shenzhen, 518055, China*

**This file includes:**

- Supporting Note 1-5
- Supporting tables S1-S9
- Supporting figures S1-S7
- Reference

**Supplementary Note 1 | Details of the partitioning behavior of Cu.**

The chemical compositions of L2_1_ and BCC phases were analyzed using EDS-Pointing analysis (**Table S1**). To further investigate elementals distribution, EDS-Mapping analysis was performed, as shown in **Fig. S2**. The mapping results indicate that Al, Ni, Ti, and Cu are enriched in the L2_1_ phase, whereas Fe and V preferentially partition to the BCC phase. The partitioning behavior of Cu can be understood through mixing enthalpy considerations. The more negative the mixing enthalpy, the stronger the atomic bonding. The mixing enthalpies of Cu with Al, Fe, V, Ni and Ti elements are −1, +13, +5, +4 and −9 kJ/mol, respectively. Therefore, As the Cu content increases from 0 to 1.0 at.%, Cu is more readily soluble in the Al, Ni and Ti enriched L2_1_ phase.

**Supplementary Note 2 | Calculation of** $\boldsymbol{\Delta}\boldsymbol{\sigma}_{\boldsymbol{ss}}$ **for multi-phase complex concentration alloys.**

The lattice distortion induced by the size and modulus mismatch between the solute atoms enhances the overall lattice distortion of the alloy, creating localized elastic stress fields. The interaction of these localized elastic stress fields and the dislocation stress field hinder dislocations movement, thereby increasing the strength of the alloy. In addition, extensive research has shown that the modulus-normalized critical resolved shear stress (CRSS)[1] is affected by the concentration of solute atoms. When the interactions among solutes are ignored, the SSH effect in concentrated binary alloy can be quantified by the Labusch model, which can be expressed as:

$\Delta\sigma_{ss}$ *=B_i_X_i_^2/3^* (1)

where the *B_i_* is:

B*_i_*=3G$\varepsilon_{i}$^4/3^; $\varepsilon_{i}=\left( \delta_{G_{i^{'}}}^{2}+\alpha^{2}\delta_{i}^{2} \right)^{1/2}$; $\delta_{G_{ij}}^{2}=\frac{\delta_{G_{ij}}}{1+\left| \delta_{G_{ij}} \right|/2}$ (2)

G is the shear modulus of the solvent element;$\alpha$ is a physical constant associated with the dislocation, whose value is 16 in HEAs[2]; Z is a constant for the single-phase refractory high entropy alloys (HEAs)[3].

For the multicomponent alloys that are short of well-defined principal elements as solvents, Gypen and Deruyttere propose an approach to quantify the solid solution hardening effect[4], which can be expressed as:

$\Delta\sigma_{ss}$ *=*$\left( \sum_{i} B^{1/n}X_{i} \right)^{n}$ (3)

where n is the concentration in the range 0.5-1.0. There are three assumed conditions for the configurations of solute atoms in solvent to establish this model, which are dilute, statistical, and interaction-free.

When we understand the solid-solution strengthening effect in HEAs, there is a key issue to simplify multi-principal elements and high concentration. Therefore, combined with Eqs. (1)-(3), the solid-solution strengthening effect in HEAs is simplified, similar to conventional solid-solution alloys, which is expressed as:

$\Delta\sigma_{ss}$ *=* $3GZ\left( \sum_{i} \varepsilon_{i}^{2}X_{i} \right)^{2/3}$ (4)

where *G* is the shear modulus of the solvent matrix, which is calculated using *G* = $\sum_{i} G_{i}X_{i}$, in which the $G_{i}$ and $X_{i}$ are the shear modulus and molar concentration of constituent elements in HEAs, respectively. After the Z value was finalized (Z value is 0.0074 ± 0.0011 with a variation of <15%, which is statistically determined)[3], and applied in Eq. (4), the *Δ*$\sigma$*_ss_* is simplified as[2]:

$\Delta\sigma_{ss}$ = *G*$\left( \sum_{i} \varepsilon_{i}^{2}X_{i} \right)^{2/3}$/45 (5)

Apart from this, the atomic size and shear modulus distortions in the HEAs should be estimated and given below:

$\delta_{i}$ = $\frac{9}{8}\sum X_{j}\delta_{ij}$ (6)

$\delta_{G_{i}^{'}}$ = $\frac{9}{8}\sum X_{j}\delta_{G_{ij}^{'}}$ (7)

where $\delta_{ij}$ = 2($r_{i}-r_{j}$) / ($r_{i}+r_{j}$) is the difference in atomic radius between elements *i* and *j*. Similarly, $\delta_{ij}$ = 2($G_{i}-G_{j}$) / ($G_{i}+G_{j}$) is the difference in shear modulus. The $r_{i}$, $r_{j}$, $G_{i}$, and $G_{j}$ are the radius and shear modulus of elements *i* and *j*, respectively, which are listed in **Table S5**. The atomic size difference $\delta_{ij}$ and modulus difference *δ_G’ij_* of the Al-Fe-Ni-Ti-V-Cu alloy element pairs are listed in **Table S6**.

The theory of solid solution strengthening of multicomponent L2_1_ phases does not exist in the literature. The solid-solution strengthening of both L2_1_ and BCC phases should be included in the calculation of the overall yield strength[2]:

$\Delta\sigma_{ss}$ = $\psi_{BCC}{\sigma_{ss}}^{BCC}$ + $\psi_{{L2}_{1}}{\sigma_{ss}}^{{L2}_{1}}$ (8)

where $\psi_{BCC}$, $\psi_{{L2}_{1}}$are the volume fractions of BCC and L2_1_ phases, respectively, as listed in **Table S7**. Based on Eq. (8), the value of solid solution strengthening effect of (Al_15_Fe_35_Ni_30_Ti_15_V_5_)_99_Cu_1_ high entropy alloy can be obtained as about 187 MPa.

**Supplementary Note 3 | Calculation of** $\boldsymbol{\Delta}\boldsymbol{\sigma}_{\boldsymbol{GB}}$ **for multi-phase complex concentration alloys.**

The grain boundary strengthening to yield strength of (Al_15_Fe_35_Ni_30_Ti_15_V_5_)_99_Cu_1_ CCA can be estimated using the following formula[5]:

*Δ*$\sigma_{GB}$*=*k*_HP_d^-^*^1/2^ (9)

The Hall-Petch coefficient, k_HP_, can be approximated using k_HP_ ≈0.42G$\sqrt{b}$[6], where the shear modulus G is 69.2 GPa, and the Burgers vector b is given as b = ((d_hkl,_ $\text{L2}_{\text{1}}$/2) + d_hkl, BCC_)/2. Using these parameters, k_HP_ was calculated to be 417.3 MPa🞌μm^1/2^. The average grain size, derived from the EBSD results (see Supplementary **Fig. S5**), for the Cu1.0 alloy is 66.8±5.0 μm, and the contribution of grain boundary strengthening to the yield strength is 51 MPa.

**Supplementary Note 4 | Calculation of** $\boldsymbol{\Delta}\boldsymbol{\sigma}_{\boldsymbol{ppt}}$ **for multi-phase complex concentration alloys.**

The strength increment of alloys from precipitates can be calculated from four possible mechanisms: 1) order strengthening, 2) modulus strengthening, 3) coherency strengthening, and 4) Orowan dislocation looping. In order to apply the reinforcement model, we assume that the shape of the precipitates is spherical in evaluating the precipitate hardening mechanism (In the case of the cuboidal precipitates in Cu0, Cu1.5 alloys, it was assumed that the width was the same as the diameter). When a dislocation passes through the ordered precipitate, an antiphase boundary with a higher energy state as compared to that of the desired bonding can be formed. Furthermore, the difference in shear modulus between the matrix and the precipitates leads to local changes in the line tension in the poorly discharged segments pass through the precipitates. Therefore, order strengthening and modulus strengthening are the controlling mechanisms during the precipitate shearing. The occurrence of dislocation shearing depends on the size of the precipitate. When the size of the precipitate is smaller than a critical radius (r_crit._), dislocation shearing occurs, while above that, dislocation looping is formed. Dislocation looping is generally caused by incoherent particles, and it is difficult for precipitates to maintain lattice coherency. However, if the size of the precipitates is large enough, dislocation cyclisation occurs despite lattice coherence. The critical radius where the transition from dislocation shearing to looping appears is defined as r*_crit._* = b/2$\delta$, where b is the Burgers vector of dislocation. As summarized in **Table S8**, the critical radius of precipitate is ~7.1 nm, indicating that both dislocation shearing and Orowan dislocation looping mechanisms are operative at room temperature. For the ~3 nm nanoprecipitates, which are smaller than the critical radius (~7.1 nm), dislocation shearing occurs during deformation. The coherent strain field around the ~100 nm precipitates bend the dislocations towards lower energy positions, thus hindering dislocation motion. The coherency strengthening, $\Delta\sigma_{coh}$, is given by the following equations[7]:

$\Delta\sigma_{coh1}=M\alpha_{\varepsilon}{(G\varepsilon)}^{3/2}{(rf/0.5Gb)}^{1/2}$ (10)

$\Delta\sigma_{coh2}=1.2Mf^{1/2}{(\frac{0.125G^{4}b^{3}\varepsilon}{r^{3}})}^{1/4}$ (11)

where $\alpha_{\varepsilon}$ (= 2.6) is a constant, $\varepsilon$is the constrained lattice misfit ($\varepsilon$ ≈ $\frac{2}{3}$ $\delta$), G is the shear modulus of matrix, and $f$ is the volume fraction of the precipitate. Eq. (11) is dominant for larger precipitates where the dislocations do not cut through the precipitates. At this time, since the movement of dislocation has to overcome the elastic interference by a large number of precipitates, the coherency strengthening is inversely proportional to the size of precipitate. Thus, Cu1.0 with the smallest precipitate size and lattice mismatch achieves the highest coherent strengthening. Similarly, the extent of strengthening via the Orowan looping is also governed by the size of precipitate, and it can be expressed using the following equation[8]:

$\Delta\delta_{OR}=\frac{0.4MGb}{\pi\lambda\sqrt{1-\nu}}\times\ln\left( \frac{1.63r}{b} \right)$ (12)

$\lambda=1.63r\times$(${\frac{\pi}{4f}}^{\frac{1}{2}}-2$) (13)

where $\lambda$ indicates the average inter-precipitate spacing.

**Supplementary Note 5 |** **Materials and methods.**

***Alloy fabrication***

Bulk CCAs ingots with the nominal compositions of (Al_15_Fe_35_Ni_30_Ti_15_V_5_)_100-x_Cu_x_ (x = 0, 0.5, 1.0, 1.5 at.%) were synthesized by arc melting. High-purity elemental metals (> 99.9 wt.%) -Al, Fe, Ni, Ti, V, and Cu-were weighed, mixed and melted in a water-cooled copper hearth using an arc melting furnace (NMS-DRⅢ, China). To minimize the volatilization of low-melting-point elements, the raw materials were placed at the bottom of the crucible during melting. All melting processes were conducted under a high-purity argon atmosphere with a vacuum level of 2.5 × 10^−3^ Pa to prevent oxidation. Each ingot was flipped and remelted five times to ensure chemical homogeneity. The final ingot weighed approximately 20 g. The alloys were designated as Cu0, Cu0.5, Cu1.0, and Cu1.5 according to their Cu content. The density of each ingot was measured using the Archimedes drainage method on a precision balance with an accuracy of 0.0001 g.

***Phase and microstructure characterization***

Phase identification was carried out by X-ray diffraction (XRD, D8 Advance, Bruker, Germany) using Cu Kα radiation over a 2*θ* range of 20° -100° at a scanning rate of 5°/min. Microstructural characterization of polished surfaces and fracture cross-sections was conducted using scanning electron microscope (SEM, Crossbeam 350, Zeiss, Germany) in backscattered electron (BSE) mode. Elemental composition and distribution were analyzed using energy-dispersive X-ray spectroscopy (EDS). Transmission electron microscope (TEM, Tecnai F30, FEI, USA) was employed to investigate the microstructure and interfacial features in greater detail. Fracture surfaces were further examined using a field-emission SEM (Merlin, Zeiss, Germany). Grain morphology and orientation were analyzed by electron backscatter diffraction (EBSD, c-nano, Oxford, U.K.) attached to the SEM.

***Mechanical property tests***

Mechanical properties were evaluated by uniaxial compression tests at room temperature (RT), as well as at elevated temperatures of 600 ℃, 700 ℃, 800 ℃, and 900 ℃. Compression specimens were prepared by wire electrical discharge machining (WEDA), with dimensions of 2 mm × 2 mm × 4 mm for RT tests and 4 mm × 4 mm × 6 mm for high-temperature tests. To minimize surface defects, all specimens were polished sequentially using 220-, 400-, 1200- and 2000-grit SiC papers. Room-temperature compression tests were performed on a universal mechanical testing machine (AGX-VD, Shimadzu, Japan) at a constant strain rate of 5 × 10^−4^ s^−1^. Interrupted compression tests at various strain levels were conducted to investigate microstructural evolution during deformation. High-temperature compression tests in the range of 600 ℃-900 ℃ were carried out on a thermo-mechanical simulator (Gleeble-3500, DSI, USA) with a quasi-static strain rate of 10^-3^ s^-1^. Each test was repeated three times to ensure the consistent data, and the average values (e.g., the stress train curves, and yield strengths) are presented.

***CALPHAD calculations***

The phase stability and solidification behavior of (Al_15_Fe_35_Ni_30_Ti_15_V_5_)_100-x_Cu_x_ (x = 0, 0.5, 1.0, 1.5 at.%) CCAs were predicted using the CALPHAD method, implemented in the Pandat software package. Vertical section phase diagrams were constructed to analyzed the solidification pathways and to assess the influence of Cu content on phase evolution.

***Statistical analysis***

All data from the repeated tests were presented as mean ± standard deviation of 3 samples. Image J software was used for quantifying the volume fractions of different phases.

**Supporting tables**

**Table S1** Chemical compositions of the (Al_15_Fe_35_Ni_30_Ti_15_V_5_)_100-x_Cu_x_ CCAs measured by EDS analysis (at.%).

| Alloys | Phase | Al | Ti | V | Fe | Ni | Cu |
| --- | --- | --- | --- | --- | --- | --- | --- |
| Cu0 | BCC | 8.99±0.41 | 16.20±2.90 | 5.67±0.60 | 41.06±4.44 | 25.27±0.22 | 0 |
|  | L2_1_ | 19.63±1.11 | 15.94±0.13 | 4.14±0.11 | 27.70±1.24 | 32.74±0.26 | 0 |
| Cu0.5 | BCC | 6.18±0.85 | 10.49±1.73 | 7.12±0.61 | 48.90±4.57 | 26.90±2.53 | 0.41±0.04 |
|  | L2_1_ | 12.58±0.50 | 18.26±0.55 | 4.35±0.38 | 27.72±1.45 | 36.28±0.85 | 0.81±0.05 |
| Cu1.0 | BCC | 6.31±0.63 | 10.06±0.85 | 7.29±0.27 | 49.49±3.15 | 25.99±1.83 | 0.86±0.11 |
|  | L2_1_ | 17.30±0.64 | 17.21±0.27 | 3.40±0.18 | 23.62±0.17 | 37.20±0.78 | 1.27±0.37 |
| Cu1.5 | BCC | 3.65±0.76 | 11.21±0.21 | 5.30±1.19 | 45.92±0.71 | 30.10±0.29 | 1.17±0.12 |
|  | L2_1_ | 8.16±1.09 | 17.04±1.19 | 5.07±0.26 | 33.11±3.58 | 34.22±2.86 | 2.43±0.54 |

**Table S2** A summary of mechanical properties at RT of (Al_15_Fe_35_Ni_30_Ti_15_V_5_)_100-x_Cu_x_ CCAs: compressive yield strength(𝜎_y_), ultimate compressive strength(𝜎_UCS_) and fracture strain (*ε_f_*) at room temperature.

| Sample | Yield  strength (MPa) | Compressive  Strength (MPa) | Plastic  Strain (%) |
| --- | --- | --- | --- |
| Cu0 | 2140.9±50.1 | 2699.7±28.0 | 6.7±0.4 |
| Cu0.5 | 2347.2±5.1 | 3145.9±10.5 | 10.4±0.3 |
| Cu1.0 | 2375.2±6.6 | 3371.6±80.4 | 11.8±0.2 |
| Cu1.5 | 2300.4±61.1 | 3145.8±111.1 | 9.6±0.9 |

**Table S3** A summary of mechanical properties at 600-900 ℃ of Cu1.0 alloy.

| Temperature (℃) | 600 | 700 | 800 | 900 |
| --- | --- | --- | --- | --- |
| Yield  strength (MPa) | 1311.6±15.2 | 901.1±18.3 | 403.5±13.1 | 177.4±8.6 |

**Table S4** Details of literature sample states, testing methods, density determination, and references for **Fig.3 (c) and (d)**.

| Sample | Sample states | Density determination | References |
| --- | --- | --- | --- |
| Al_17_Ni_34_Ti_17_V_32_ | As-cast | Archimedes method | [9] |
| AlCrFeNi | Homogenized | Volume method | [10] |
| (AlCrFeNi)_88_Ti_12_ | Homogenized |  |  |
| (AlCrFeNi)_88_V_12_ | Homogenized |  |  |
| (AlCrFeNi)_88_Ti_6_V_6_ | Homogenized |  |  |
| (AlCrFeNi)_97_Mo_3_ | As-cast | Not mentioned | [11] |
| Al_2.7_TiVCrCu-cast | As-cast | Not mentioned | [12] |
| Al_2.7_TiVCrCu-annealing | Annealed |  |  |
| Al_0.75_CrFeNi | As-cast | Not mentioned | [13] |
| AlNbTiV | Homogenized | Hydrostatic weighting | [14] |
| Al_0.5_Ti_2_Nb_1_Zr_1_W_x_ | Homogenized | Archimedes method | [15] |
| AlTiVCoNi | Annealed | Archimedes method | [16] |
| AlCr_1.3_TiNi_2_-cast | As-cast | Archimedes method | [17] |
| Ti_2_ZrHf_0.5_VNb_x_ | As-cast | Archimedes method | [18] |
| Al_0.8_Nb_0.5_Ti_x_V_2_Zr_0.5_ | As-cast | Theoretical calculation | [19] |
| AlCrFeNiTi_x_ | As-cast | Archimedes method | [20] |
| Al_15_Fe_40-x_Ni_30_Ti_15_V_x_ | As-cast | Archimedes method | [21] |
| TiZrHf_0.5_VNb_0.5_Al_x_ | As-cast | Archimedes method | [22] |
| Al_x_Cr_5_Fe_50_Mn_40-x_Ti_5_ | As-cast | Theoretical calculation | [23] |
| Al_0.5_NbTi_3_V_x_Zr_2_ | As-cast | Archimedes method | [24] |

**Table S5** Atomic radius, *r* = $\frac{\sqrt{\text{3}}}{\text{4}}a$, shear modulus, *G* of relevant pure elements in AlFeNiTiVCu alloy.

| Properties | Elements | | | | | |
| --- | --- | --- | --- | --- | --- | --- |
|  | Ni | Al | Ti | Fe | V | Cu |
| *r* (pm) | 124.0 | 143.0 | 141.8 | 126.0 | 134.0 | 127.8 |
| *G* (GPa) | 80.0 | 25.0 | 45.6 | 81.4 | 47.0 | 46.8 |

**Table S6** Relative atomic size different, (underlined numbers), and modulus different, (bold numbers) of the AlFeNiTiVCu alloy element pairs.

| Elements i/j $\delta_{ij}$/***δ_G’ij_*** | Ni | Al | Ti | Fe | V | Cu |
| --- | --- | --- | --- | --- | --- | --- |
| Ni | 0 | **1.05** | **0.55** | **-0.02** | **0.52** | **0.56** |
| Al | 0.14 | 0 | **-0.58** | **-1.06** | **-0.61** | **-0.57** |
| Ti | 0.13 | -0.01 | 0 | **-0.56** | **-0.03** | **0.01** |
| Fe | 0.02 | -0.13 | -0.12 | 0 | **0.54** | **0.58** |
| V | 0.08 | -0.06 | -0.06 | 0.06 | 0 | **0.04** |
| Cu | 0.03 | -0.11 | -0.10 | 0.01 | -0.05 | 0 |

**Table S7** Volume fractions (*f*)of BCC phase and L2_1_ precipitated phase in the as-cast (Al_15_Fe_35_Ni_30_Ti_15_V_5_)_99_Cu_1_ alloy.

| Phases | *f* (%) |
| --- | --- |
| BCC | 45.4 |
| L2_1_ | 54.6 |

**Table S8** A summary of the critical radius r*_crit._* where the transition from precipitate shearing to dislocation looping occurs.

| Alloy | r*_crit._* (nm) |
| --- | --- |
| Cu0 | 6.4 |
| Cu0.5 | 6.8 |
| Cu1.0 | 7.1 |
| Cu1.5 | 6.6 |

**Table S9** Lattice constants and the calculated lattice misfit between the L2_1_ and BCC phases in the Cu1.0 alloy after high-temperature compression at 600 and 900 ℃, as determined by HRTEM.

| Alloys | HRTEM $\text{(110)}_{\text{BCC}}$ & $\text{(220)}_{\text{L2}_{\text{1}}}$ diffraction | | |
| --- | --- | --- | --- |
|  | $a_{BCC}(Å)$ | $a_{{L2}_{1}}(Å)$ | $\text{δ}_{\text{HR}}\text{(\%)}$ |
| 600 ℃ | 2.945±0.006 | 5.994±0.011 | 1.77 |
| 900 ℃ | 2.951±0.002 | 6.054±0.077 | 2.57 |

**Supporting figures**

**
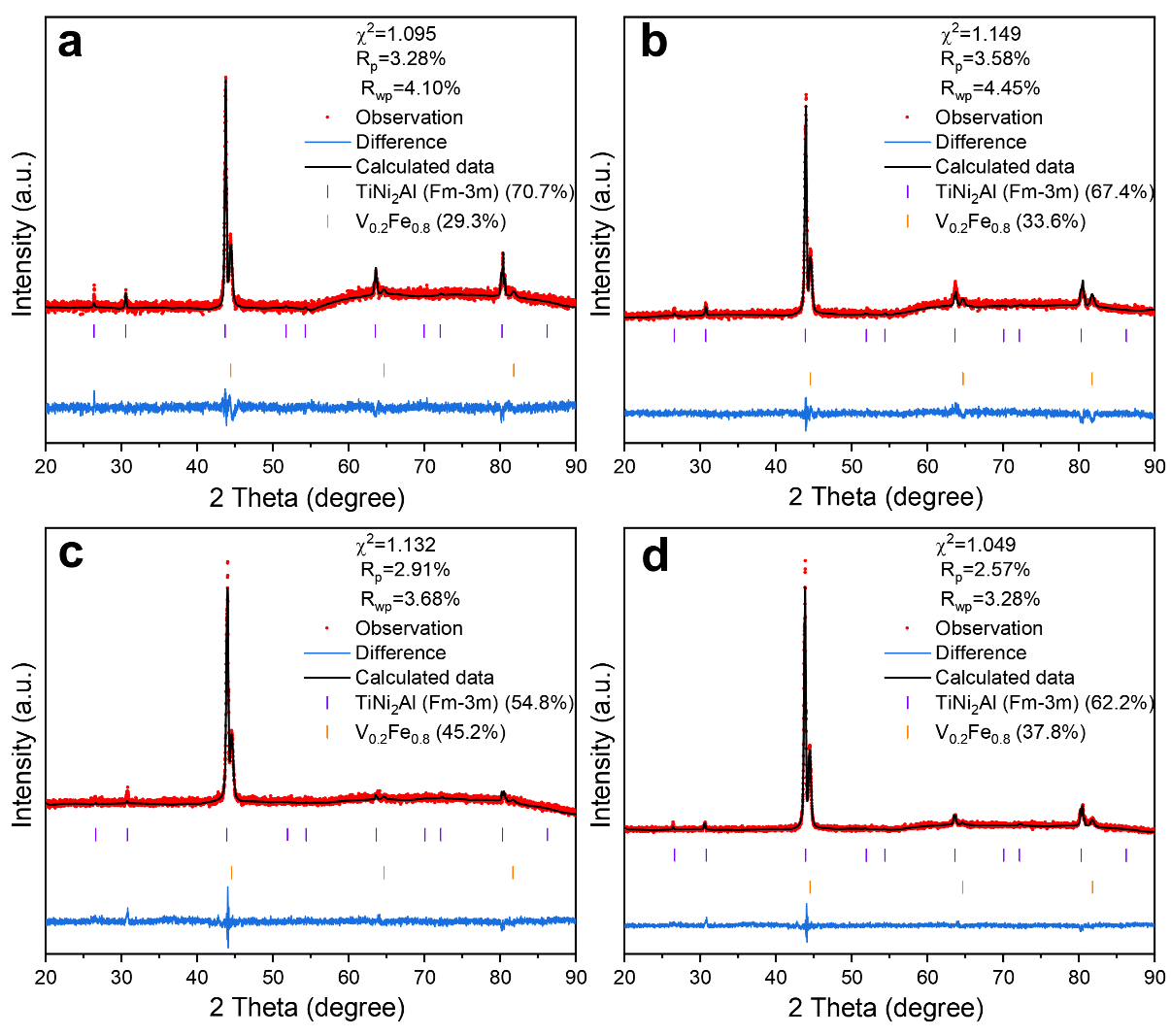
**

**Fig. S1** The output from the Rietveld refinement analysis of the XRD pattern for (Al_15_Fe_35_Ni_30_Ti_15_V_5_)_100-x_Cu_x_ CCAs: (a) Cu0, (b) Cu0.5, (c) Cu1.0, (d) Cu1.5.

**
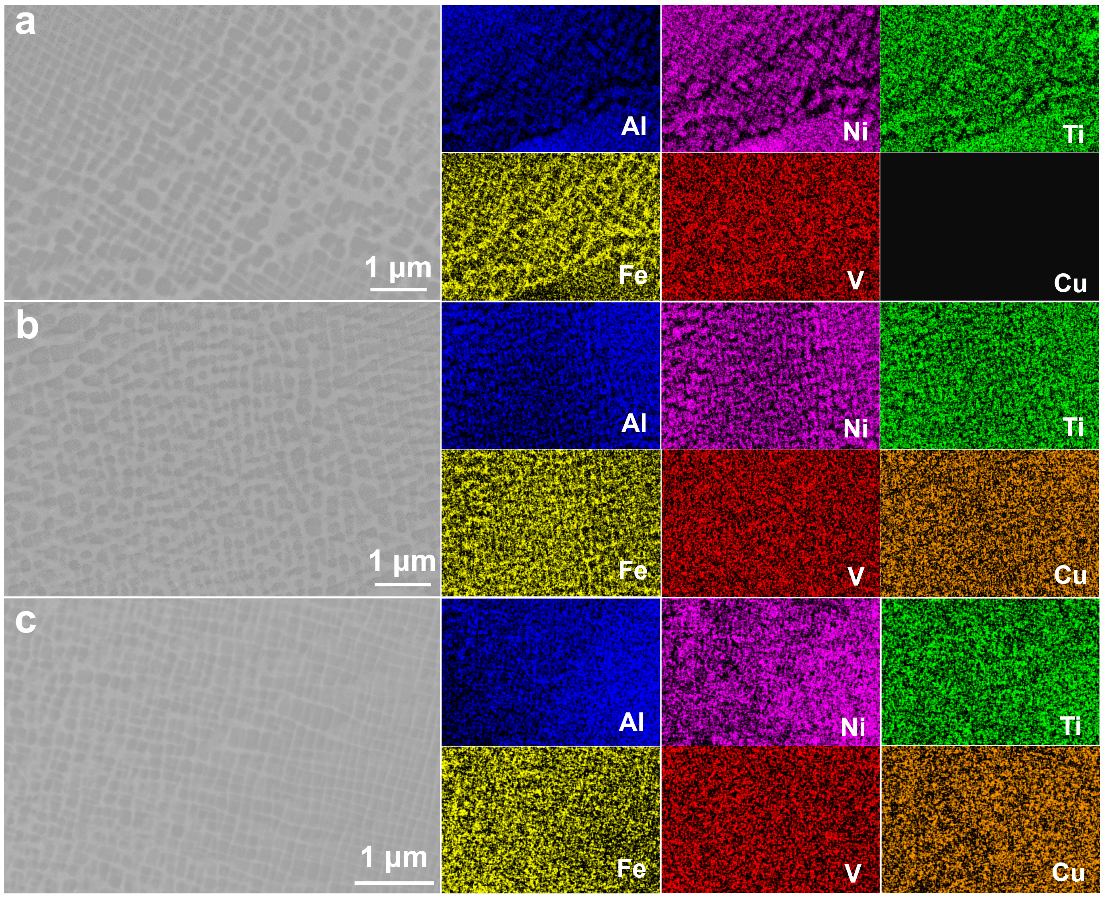
**

**Fig. S2** EDS mapping of the (Al_15_Fe_35_Ni_30_Ti_15_V_5_)_100-x_Cu_x_ CCAs: (a) Cu0, (b) Cu0.5, (c) Cu1.0.

**
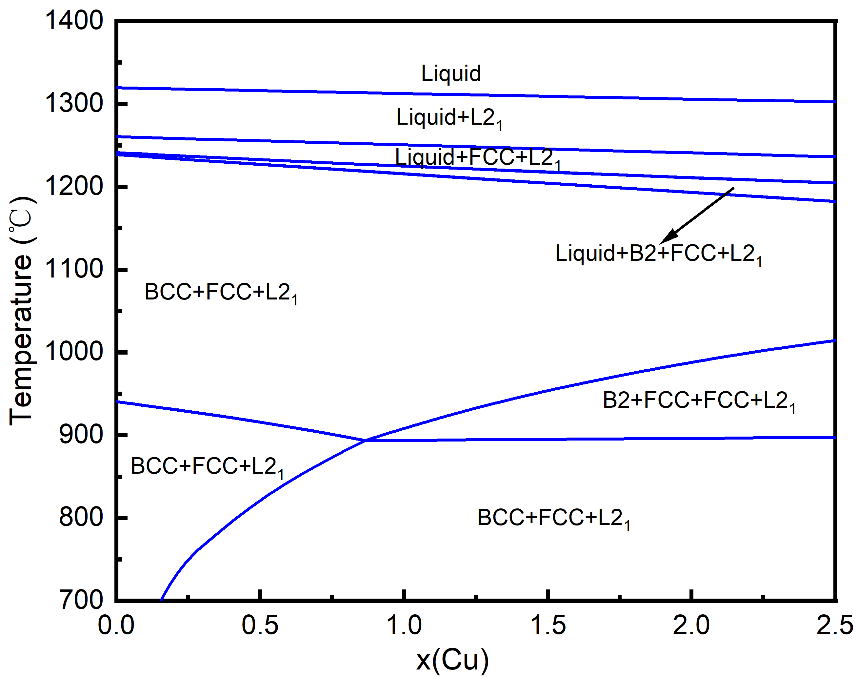
**

**Fig. S3** Vertica cross-section of the phase diagram of (Al_15_Fe_35_Ni_30_Ti_15_V_5_)_100-x_Cu_x_ CCAs.

**
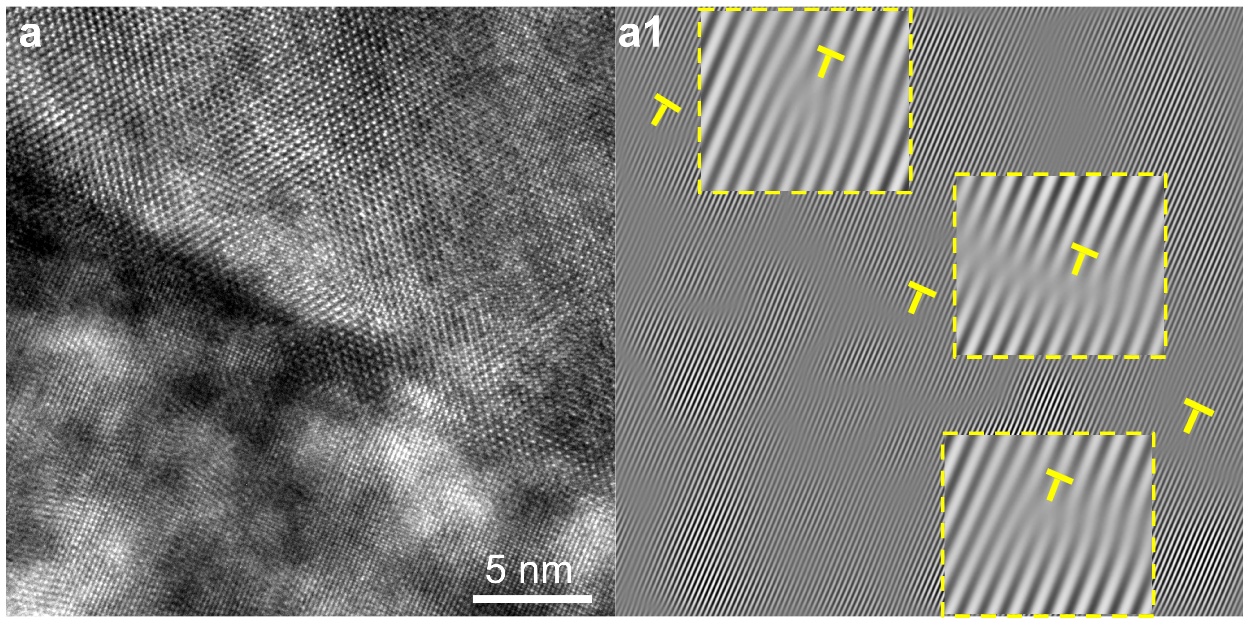
**

**Fig. S4** The HRTEM image and the corresponding inverse FFT of Cu0, showing the dual-phase interface of L2_1_/BCC: (a) HRTEM images of the alloy; (b) the corresponding inverse FFT.


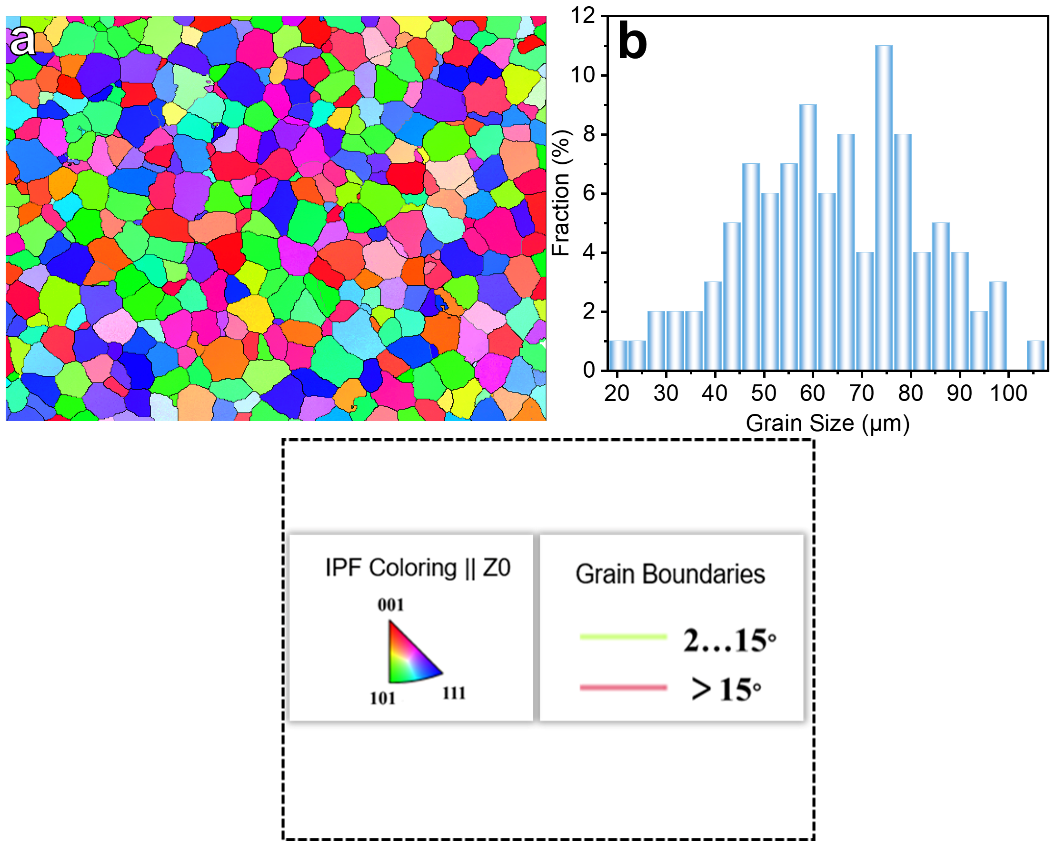


**Fig. S5** (a) Inverse pole figure (IPF), (b) grain size distribution of the (Al_15_Fe_35_Ni_30_Ti_15_V_5_)_99_Cu_1_ CCAs.


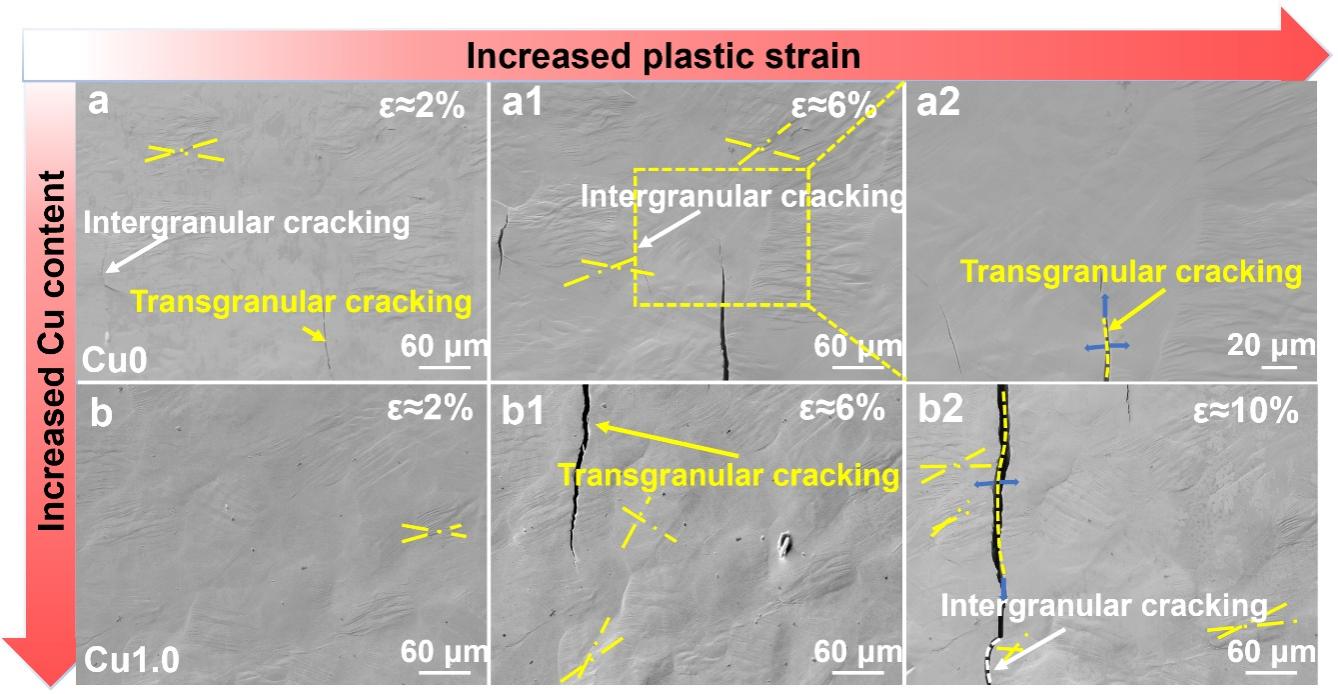


**Fig. S6** Quasi-in-situ SEM images show that the plastic deformation of Cu0/Cu1.0 increases with increasing macroscopic strain: (a-a1) Cu0 at 2% and 6% strain, respectively; (a2) an enlarged view of (a1); (b-b2) Cu1.0 at 2%, 6% and 10% strain, respectively.

**
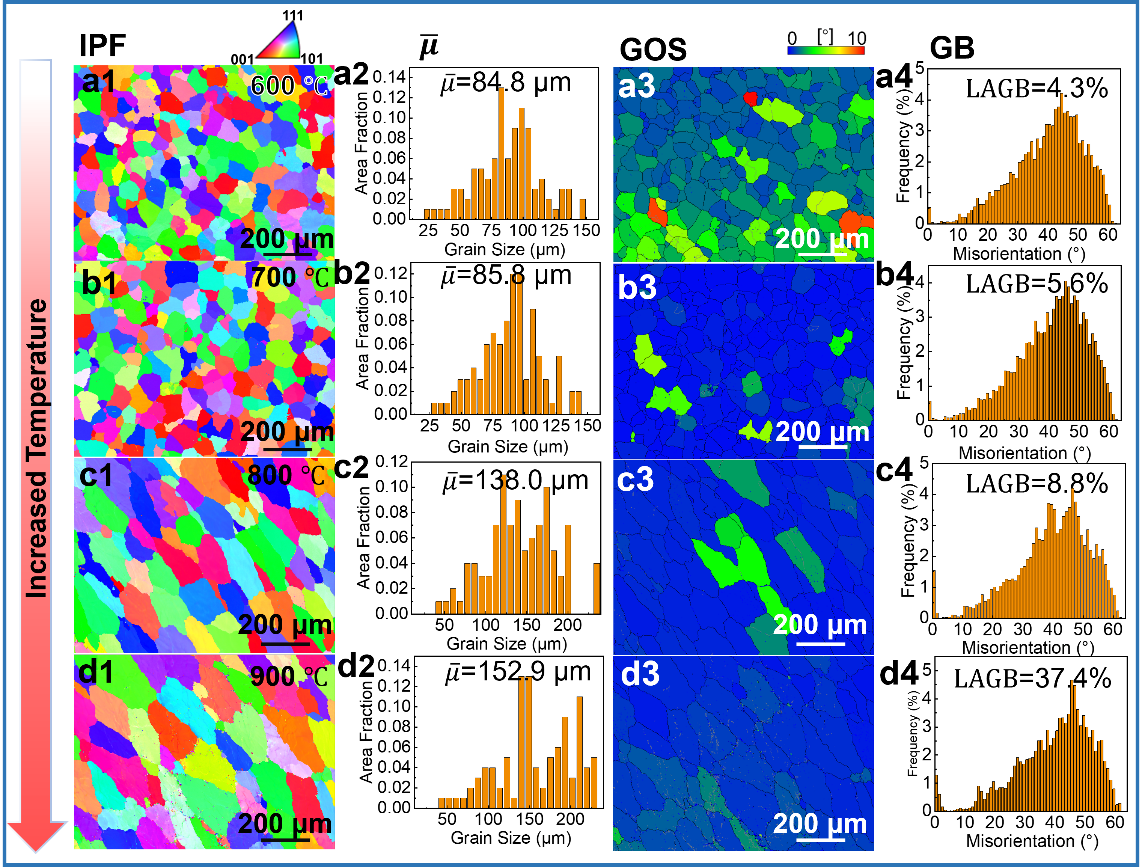
**

**Fig. S7** Inverse pole figure (IPF), grain size distribution, grain orientation spread (GOS) and disorientation angle distribution of Cu1.0 alloy deformed at elevated temperature: (a1-a4) 600 ℃, (b1-b4) 700 ℃, (c1-c4) 800 ℃, (d1-d4) 900 ℃.

**Reference**

[1] B. Wei, W. Wu, M. Gong, et al., Influence of lowering basal stacking fault energy on twinning behaviours, Acta Materialia 245 (2023) 118637. https://doi.org/10.1016/j.actamat.2022.118637.

[2] T. Xiang, P. Du, Z. Cai, et al., Phase-tunable equiatomic and non-equiatomic Ti-Zr-Nb-Ta high-entropy alloys with ultrahigh strength for metallic biomaterials, Journal of Materials Science & Technology 117 (2022) 196–206. https://doi.org/10.1016/j.jmst.2021.12.014.

[3] Y.S. Kim, R. Ozasa, K. Sato, O. Gokcekaya, T. Nakano, Design and development of a novel non-equiatomic Ti-Nb-Mo-Ta-W refractory high entropy alloy with a single-phase body-centered cubic structure, Scripta Materialia 252 (2024) 116260. https://doi.org/10.1016/j.scriptamat.2024.116260.

[4] L.A. Gypen, A. Deruyttere, Multi-component solid solution hardening - Part 2 Agreement with experimental results, Journal of Materials Science 12 (1977) 1034–1038. https://doi.org/10.1007/BF00540988.

[5] A. Watanabe, T. Yamamoto, Y. Takigawa, Tensile strength of nanocrystalline FeCoNi medium-entropy alloy fabricated using electrodeposition, Scientific Reports 12 (2022) 12076. https://doi.org/10.1038/s41598-022-16086-6.

[6] Z.C. Cordero, B.E. Knight, C.A. Schuh, Six decades of the Hall-Petch effect - a survey of grain-size strengthening studies on pure metals, International Materials Reviews 61 (2016) 495–512. https://doi.org/10.1080/09506608.2016.1191808.

[7] W.C. Kim, M.Y. Na, H.J. Kwon, et al., Designing L2_1_-strengthened Al-Cr-Fe-Ni-Ti complex concentrated alloys for high temperature applications, Acta Materialia 211 (2021) 116890. https://doi.org/10.1016/j.actamat.2021.116890.

[8] S. Peng, Z. Wang, J. Li, Q. Fang, Y. Wei, Beyond Orowan hardening: Mapping the four distinct mechanisms associated with dislocation-precipitate interaction, International Journal of Plasticity 169 (2023) 103710. https://doi.org/10.1016/j.ijplas.2023.103710.

[9] M. Wang, Y. Lu, J. Lan, et al., Lightweight, ultrastrong and high thermal-stable eutectic high-entropy alloys for elevated-temperature applications, Acta Materialia 248 (2023) 118806. https://doi.org/10.1016/j.actamat.2023.118806.

[10] M. Wang, Z. Wen, L. Liang, et al., Excellent combination of compressive strength and strain of AlCrFeNi MPEAs via adding Ti and V, Journal of Alloys and Compounds 947 (2023) 169560. https://doi.org/10.1016/j.jallcom.2023.169560.

[11] E. Jumaev, M.A. Abbas, S.C. Mun, G. Song, S.J. Hong, K.B. Kim, Nano-scale structural evolution of quaternary AlCrFeNi based high entropy alloys by the addition of specific minor elements and its effect on mechanical characteristics, Journal of Alloys and Compounds 868 (2021) 159217. https://doi.org/10.1016/j.jallcom.2021.159217.

[12] K. Liu, J. Wang, X. Li, Q. Qin, S. Wu, H. Yu, A new lightweight Al_2.7_TiVCrCu high entropy alloy with excellent strength and toughness after homogenization treatment, Materials Science and Engineering: A 869 (2023) 144779. https://doi.org/10.1016/j.msea.2023.144779.

[13] W. Jiao, T. Li, X. Chang, et al., A novel Co-free Al_0.75_CrFeNi eutectic high entropy alloy with superior mechanical properties, Journal of Alloys and Compounds 902 (2022) 163814. https://doi.org/10.1016/j.jallcom.2022.163814.

[14] N.D. Stepanov, D.G. Shaysultanov, G.A. Salishchev, M.A. Tikhonovsky, Structure and mechanical properties of a light-weight AlNbTiV high entropy alloy, Materials Letters 142 (2015) 153–155. https://doi.org/10.1016/j.matlet.2014.11.162.

[15] M. Abubaker Khan, T.L. Wang, C. Feng, et al., A superb mechanical behavior of newly developed lightweight and ductile Al_0.5_Ti_2_Nb_1_Zr_1_W_x_ refractory high entropy alloy via nano-precipitates and dislocations induced-deformation, Materials & Design 222 (2022) 111034. https://doi.org/10.1016/j.matdes.2022.111034.

[16] Y. Li, W.B. Liao, H. Chen, et al., A low-density high-entropy dual-phase alloy with hierarchical structure and exceptional specific yield strength, Science China Materials 66 (2023) 780–792. https://doi.org/10.1007/s40843-022-2178-x.

[17] M. Wang, Y. Lu, T. Wang, et al., A novel bulk eutectic high-entropy alloy with outstanding as-cast specific yield strengths at elevated temperatures, Scripta Materialia 204 (2021) 114132. https://doi.org/10.1016/j.scriptamat.2021.114132.

[18] D.X. Qiao, H. Jiang, W.N. Jiao, Y.P. Lu, Z.Q. Cao, T.J. Li, A novel series of refractory high-entropy alloys Ti_2_ZrHf_0.5_VNb_x_ with high specific yield strength and good ductility, Acta Metallurgica Sinica (English Letters) 32 (2019) 925–931. https://doi.org/10.1007/s40195-019-00921-3.

[19] Y. Zhao, L. Sun, L. Wang, J. Zhang, Y. Fang, X. Zhan, Synergistic strength-ductility enhancement of AlNbTiVZr lightweight refractory high entropy alloys with regulated Laves distribution morphology: Network-to-dispersion transformation, Materials Science and Engineering: A 927 (2025) 148001. https://doi.org/10.1016/j.msea.2025.148001.

[20] J.Z. Li, W.Z. Bao, J. Chen, et al., Design and fabrication of lightweight AlCrFeNiTi_x_ compositionally complex alloys with exceptional specific strength, Rare Metals 43 (2024) 3314–3328. https://doi.org/10.1007/s12598-024-02657-1.

[21] H.M. Chen, Z.Y. Cai, J. Chen, et al., Tailoring L2_1_ strengthening in lightweight AlFeNiTiV complex concentrated alloys for elevated-temperature applications, Rare Metals 44 (2025) 3479-3495. https://doi.org/10.1007/s12598-025-03241-x.

[22] D. Qiao, H. Liang, S. Wu, et al., The mechanical and oxidation properties of novel B2-ordered Ti_2_ZrHf_0.5_VNb_0.5_Al_x_ refractory high-entropy alloys, Materials Characterization 178 (2021) 111287. https://doi.org/10.1016/j.matchar.2021.111287.

[23] R. Feng, C. Zhang, M.C. Gao, et al., High-throughput design of high-performance lightweight high-entropy alloys, Nature Communications 12 (2021) 4329. https://doi.org/10.1038/s41467-021-24523-9.

[24] T. Wang, W. Jiang, X. Wang, et al., Microstructure and properties of Al_0.5_NbTi_3_V_x_Zr_2_ refractory high entropy alloys combined with high strength and ductility, Journal of Materials Research and Technology 24 (2023) 1733–1743. https://doi.org/10.1016/j.jmrt.2023.03.103.
